# Pregnancy Downregulates Plasmablast Metabolic Gene Expression Following Influenza Without Altering Long-Term Antibody Function

**DOI:** 10.3389/fimmu.2020.01785

**Published:** 2020-08-14

**Authors:** Dominika Swieboda, Elizabeth Q. Littauer, Jacob T. Beaver, Lisa K. Mills, Katherine M. Bricker, E. Stein Esser, Olivia Q. Antao, Dahnide T. Williams, Ioanna Skountzou

**Affiliations:** Department of Microbiology and Immunology and Emory Vaccine Center, Emory University School of Medicine, Atlanta, GA, United States

**Keywords:** influenza, pregnancy, immunology, hormones, cellular immunity, humoral immunity, B cell, metabolism

## Abstract

While the majority of influenza-infected individuals show no or mild symptomatology, pregnant women are at higher risk of complications and infection-associated mortality. Although enhanced lung pathology and dysregulated hormones are thought to underlie adverse pregnancy outcomes following influenza infection, how pregnancy confounds long-term maternal anti-influenza immunity remains to be elucidated. Previously, we linked seasonal influenza infection to clinical observations of adverse pregnancy outcomes, enhanced lung and placental histopathology, and reduced control of viral replication in lungs of infected pregnant mothers. Here, we expand on this work and demonstrate that lower infectious doses of the pandemic A/California/07/2009 influenza virus generated adverse gestational outcomes similar to higher doses of seasonal viruses. Mice infected during pregnancy demonstrated lower hemagglutination inhibition and neutralizing antibody titers than non-pregnant animals until 63 days post infection. These differences in humoral immunity suggest that pregnancy impacts antibody maturation mechanisms without alterations to B cell frequency or antibody secretion. This is further supported by transcriptional analysis of plasmablasts, which demonstrate downregulated B cell metabolism and post-translational modification systems only among pregnant animals. In sum, these findings corroborate a link between adverse pregnancy outcomes and severe pathology observed during pandemic influenza infection. Furthermore, our data propose that pregnancy directly confounds humoral responses following influenza infection which resolves post-partem. Additional studies are required to specify the involvement of plasmablast metabolism with early humoral immunity abnormalities to best guide vaccination strategies and improve our understanding of the immunological consequences of pregnancy.

## Introduction

The normal response to influenza A infection ranges from mild to asymptomatic; indeed, a serosurveillance study of volunteers who tested positive for antibodies against H1N1 revealed that the majority did not experience any symptoms (1). However, studies dating back to the 1918 pandemic suggest that pregnancy increases influenza-associated morbidity and mortality, with pregnant women at risk for developing severe influenza complications (2). Indeed, this pattern holds for all the major recent pandemics including 1918 (Spanish flu), 1957 (Asian flu), and 2009 (H1N1/2009) and to a lesser degree, for seasonal flu (2, 3). Pregnant women with seasonal influenza are 3 to 4 times more likely to die from influenza-related illness during the third trimester than non-pregnant women (3). Maternal influenza is associated with increased risk of miscarriage, preterm or small-for-gestational-age infants, and fetal death (2). Moreover, influenza infections early in gestation are linked to defects in fetal CNS development (4) and increased risk for autism, schizophrenia, neurosensory deficits, and psychosis in adult life (5). While influenza A virus has been detected in the placenta and amniotic fluid in both fatal (6, 7) and non-fatal (8) cases, there are few case reports of fetal human influenza (6, 9) and direct fetal infection (10). Swine and mouse studies suggest transplacental infection from seasonal influenza viruses is rare (11, 12). Mouse models of pregnancy and influenza have shown that infection during gestation has a detrimental effect on neonatal growth and development (12, 13). These findings are similar to the higher maternal mortality seen in human pregnancies during previous pandemics (14).

The pathogenesis of a complex disease like influenza likely involves a combination of direct virus effects in the respiratory compartment and an imbalance between the beneficial and harmful effects of immune mediators (15). An immune response to influenza infection requires robust production of interferons and an innate response by neutrophils, macrophages and dendritic cells resulting in activation of CD4^+^ and CD8^+^ T cells (2); however, the primary mechanisms of influenza pathogenesis are direct lung infection and subsequent compromise of lung's physiology due to infection of the respiratory epithelium, combined with the results of lung inflammation by the host's attempt to contain the virus (16).

Importantly, the immunological changes occurring during pregnancy are theoretically compatible with increased risk or severity of certain infections, including influenza. Human pregnancy is associated with changes of innate immunity such as increases in phagocytic cell numbers (17), phagocytic activity, and circulating PMNs (18), decreased numbers of plasmacytoid dendritic cells (pDCs) (19), and reductions in antigen-presenting capacities of dendritic cells (DCs) and macrophages (20). This may be further complicated by alterations in DC phenotype resulting in elevated IL-6, IL-12, and TNF-α (21). Dysregulation of innate immune responses involving TNF-α has been linked to severe influenza (22). PBMCs from pregnant women have attenuated antiviral immune responses, with reduced production of IFN-α and IFN-λ (23). Indeed, altered cytokine production, lung DC function and IFN signaling (24) have been implicated in impaired adaptive responses, impaired viral clearance, and exaggerated innate immune responses in mouse models of influenza infection. Therefore, the maternal immune response may sacrifice efficient viral clearance in favor of protecting the fetus by specifically limiting inflammatory cytokines, such as IFN-y and VEGF, in favor of increasing phagocytic cell recruitment and activity through enhanced TNFa and G-CSF production. Attempts to protect the fetus from inflammatory signals can ultimately lead to increased maternal morbidity and mortality.

Intriguingly, studies involving pregnant mice demonstrated that while individual expression of estrogen or progesterone may limit inflammation, the condition of pregnancy results in elevated inflammatory responses to influenza virus compared to non-pregnant female mice (25). Progesterone and glucocorticoids can have anti-inflammatory effects (26) which may explain the increased severity of infectious agents requiring prompt inflammatory responses for initial control and clearance, such as influenza virus (14, 27). Progesterone stimulates production of PIBF, which in turn promotes differentiation of CD4^+^ T cells into Th2 helper cells, corresponding with reductions in Th1 responses systemically and at the fetal-maternal interphase (25, 27–30). Estrogen induces less efficient APC activation, downregulates antiviral responses (including production of IFN-a) (31), and reduces CD8^+^ T cell cytotoxic activity and NK cell responses, with secretion of cytokines significantly impaired (17, 32). As NK cells and CD8^+^ T cells function to kill virus-infected cells, this may account for the delayed viral clearance observed in pregnancy. Additional physiologic changes of pregnancy, including reduced lung capacity and increased oxygen consumption (33) provide added mechanisms of enhanced risk.

The confounder of pregnancy has therefore been well established to adversely alter the clinical course of influenza (34) with short term complications to mother and baby apparent, and long-term consequences to the fetus hypothesized. However, how the condition of pregnancy impacts long-term maternal anti-influenza immunity is contentious. Published research on adaptive immune responses in animal models of influenza during pregnancy is limited. Rising estrogen concentrations reduce B cell lymphopoiesis in mice during pregnancy (35), and it has been reported that influenza-infection-induced antibody titers are lower in pregnant mice (36), although Marcelin et al. report antibody levels are comparable between pregnant and non-pregnant mice in the short term (37). High doses of estradiol stimulate antibody production in rhesus macaques (38) and augment Th2 response and humoral immunity (39), but estriol, a dominant pregnancy hormone, increases production of IgM, not IgG, in mice (40); intriguingly, patients with severe influenza are likely to be deficient in subclasses of IgG (41, 42). Zheng et al. reported significant association between imbalanced anti-H1N1 immunoglobulin subclasses and dysregulated cytokines in pregnant women hospitalized with 2009 H1N1 (43). Moreover, while there is a plethora of data on the clinical efficacy of influenza vaccines in pregnant women, there is limited details on the immunological responses to influenza immunization (2, 44). Some studies report equivalent seroconversion rates (45, 46) while others identify reduced antibody neutralization and titers for some influenza strains (47). However, to these authors knowledge, no studies have evaluated the impact of pregnancy on B cell quality and antibody secretion, or the long-term antibody characteristics, following influenza infection. Therefore, here we build on our previous study of seasonal H1N1 A/Brisbane/59/2007 in mouse pregnancy by evaluating the long-term consequences of H1N1 A/California/07/2009 infection in pregnant mice. We systematically evaluate compartment-specific pathogenesis following infection and expand upon studies of innate immunity by following cellular and humoral responses after completion of pregnancy and weaning. Importantly, we explore mechanisms of acutely delayed antibody hemagglutinin inhibition and virus neutralization through transcriptional studies of splenic plasmablasts derived from pregnant mice.

## Materials and Methods

### Animal Infections

Animal studies were conducted according to Emory University Institutional Animal Care and Use Committee (IACUC) guidelines outlined in an approved protocol (PROTO201800113) in compliance with the United States Federal Animal Welfare Act (PL 89–544) and subsequent amendments. Timed pregnancies were generated in 8- to 12-week old female BALB/c mice (Envigo; Huntingdon, United Kingdom) as described previously (12). On day 12 of pregnancy (determined by 20% weight gain and/or presence of a copulation plug), pregnant mice and non-pregnant controls were lightly anesthetized with isoflurane and infected intranasally with either 30 μl 0.5 × LD_50_ of mouse adapted A/California/07/2009, approximately 30 plaque forming units (p.f.u.) or 30 μl 2 × LD_50_ of mouse adapted A/California/07/2009, approximately 120 p.f.u. (GenBank: HA, MH429787; M2, MH429788; NA, MH429789; NP, MH429790; NS1, MH429791; PA, MH429792; PB1, MH429793; PB2, MH429794). Non-infected mice were intranasally dosed with a dilution of BALB/c lung lysate in sterile PBS.

Mice were bled 4, 7, 14, 42, 62, and 84 days post-infection. Blood was collected submandibularly, and sera was isolated and stored in 1x Halt Protease Inhibitor (ThermoFisher;Waltham, Massachusetts) at −20°C. Mice were euthanized for organ collection at 0, 2, 4, 7, 10, 14, and 42 days post-infection. Lung and placental samples were weighed, homogenized into 1x DMEM (ThermoFisher) /1 × Halt Protease Inhibitor, and stored at −80°C for western blots. Left lung lobes were homogenized in serum-free DMEM and stored in 1x Halt Protease Inhibitor (ThermoFisher) for viral titers. Viral titers were determined by plaque assay in MDCK cells (American Type Culture Collection; Manassas, Virginia) as described previously (48). Leukocytes were isolated from lungs, spleen, placenta, uterus and mediastinal lymph nodes using Histoplaque-1119 (Sigma Aldrich; St. Louis, MO). ELISPOTs and FACS staining were performed immediately.

### Histology

Organs were isolated at the indicated days post infection and submerged in histology cassettes in 4% paraformaldehyde overnight at 4°C. Tissues were embedded in paraffin, sectioned in 4 μm, and fixed to glass microscopy slides. Hematoxylin and eosin staining was performed by Yerkes National Primate Research Center Pathology Core, and slides were imaged on a Zeiss Axioscope with SpotFlex 15.2 camera with Spot Advanced 4.7 software.

### Cytokine and Hormone Quantification in the Lungs, Sera, and Placenta

Cytokines were quantified in sera, lungs, and placentas at days 4 and 7 post-infection using Bio-Rad 23-plex Mouse Group I Cytokine Assay Kit per manufacturer's instructions (Bio-Rad; Hercules, CA). Progesterone and prostaglandin F2α (PGF2α) were quantified with hormone ELISA kits (ALPCO; Salem, NH). Cytokine and hormone expression values were normalized by dilution and tissue lysate concentration. Fold changes were plotted in heat maps, and raw values plotted and reported in Supplementary Tables. Fold change was transformed as follows to aid in visualization of decreases: if fold change >1, no transformation; if fold change <1, –(10 | log10fold change |). For example, a 2-fold increase will remain +2, while a fold change of 0.5 will become −2.

### MMP, COX-2, and PIBF Quantification in Tissue Lysates

Molecular analysis of protein expression was performed on 5 mg of tissue lysate via Western blot. Purified anti-murine MMP-9 (BioLegend 819701, 1:500) and anti-murine MMP-2 (BioLegend 680002, 1:500) were detected with goat anti-rabbit (1:10,000) antibody. Rabbit anti-murine COX-2 (Abcam, ab52237) and rabbit anti-murine C13orf24 (PIBF) (Abcam, ab156267) were detected with goat anti-rabbit (1:10,000) antibodies. Rabbit anti-murine β-actin (1:2,000) was used as a loading control with goat anti-rabbit, (1:10,000) secondary antibodies. Blots were developed with Super Signal Femto Maximum Sensitivity Substrate (Thermo Scientific 34096) and imaged using a Bio-Rad ChemiDoc Touch. Volume intensity of signal was normalized to β-actin loading controls. Representative western blots can be found in Supplementary Figure 3 (for lung) and Supplementary Figure 4 (for placenta).

### Antibody Characterization and Binding Studies

Antibody titers were determined via ELISA with biotinylated anti-IgG, IgM, IgA antibodies (Southern Biotech; Birmingham, AL) against recombinant hemagglutinin H1N1 (rH1) A/California/07/2009 (NR-13691, BEI Resources (ATCC); Manassas, VA) as described previously (49). Hemagglutination inhibition (HAI) assays were performed with PBS washed turkey red blood cells in accordance with the WHO Laboratory Diagnostics Manual (50).

### Innate Immune Cell and Germinal Center Activation

Single cell suspensions were obtained from blood, spleen, liver, uterus, placenta, and lung-draining mediastinal lymph nodes (MLN) 2 days after infection. For innate immune cell analysis, cells were stained with anti-CD11c (N418), -CD11b (M1/70), -Ly6G (1A8), -F4/80 (BM8), -CD45 (30-F11), -IA/IE (M5/114.15.2), -CD64 (C54-5/7.1), and -CD24 (30-F1) (BioLegend; San Diego, California). Dead cells were excluded by gating out cells positive for Live/Dead fixable dead stain (eBioscience; San Diego, California). Following fixation in 2% paraformaldehyde, samples were acquired with an LSRII (BD Biosciences; San Jose, California) and analyzed using FlowJo v 9 (FlowJo LLC, BD; Franklin Lakes, NJ). Gating methodologies are depicted in Supplemental Figure 2.

### Quantification of Antibody and Cytokine Secreting

To quantify antibody-secreting cells (ASCs), polyvinylidene fluoride ELISpot plates (EMD Millipore, Burlington, MA) were coated with 200 ng/well rH1. Splenocytes and lung lymphocytes (1 × 10^6^ cells/well) were incubated at 37°C for 16 h. Influenza-specific antibodies were detected using isotype-specific, biotinylated murine Ig antibodies (IgA, IgM, and IgG; Southern Biotech; Birmingham, AL). To detect cytokine secreting cells, 5 × 10^5^ cells/well were overlaid in ELISPOT plates (EMD Millipore; Burlington, MA) coated with 100 ng/well of capture antibody (BD Biosciences; San Jose, CA). Cells were stimulated with 200 ng/well rH1 for 48 h at 37°C. The cells were washed and incubated with 100 ng/well biotinylated detection antibodies (IL-4, IL-10, IFN-γ, BD Biosciences) and developed with streptavidin-HRP and diaminobenzidine. Plates were analyzed via ImmunoSpot Reader 5.0 (Cellular Technology Limited**;** Shaker Heights, OH**)** and normalized to 1 × 10^6^ cells/well.

### Differential Gene Expression Analysis

RNA sequencing was performed by the Yerkes National Primate Research Center, Non-human Primate Genomics Core. 10,000 plasmablasts (CD138^+^B220^int^) were sorted from the spleens of pregnant and non-pregnant influenza virus infected mice at 10 days post infection. Single-end RNA sequencing was performed on samples in duplicate to increase sequencing depth (101 base pair reads). Sequencing was checked for quality using FastQC (Barbaham Institute, United Kingdom). Processing of single end reads was performed within the R programming language. The Rsubread package align function (51) was used to align single end reads to the *Mus musculus* genome (GRCm38.93). BAM files of duplicate samples were merged prior to the counting of reads for each gene using the featureCounts function of the Rsubread package. Differential gene expression analysis was performed in R using the DESeq2 package (52). Gene set enrichment analysis (53) was performed as previously described in pre-ranked mode with the clusterProfiler package (54).

### Statistical Analysis

All analysis, graphing, and annotation was performed in GraphPad Prism 8 (San Diego, CA). “n” represents the number of mice used for each experiment. Weights were analyzed using two-way ANOVA with Tukey post-host corrections. Survival data were analyzed using Mantel-Cox test and gestation length data were analyzed using a Kruskal-Wallis test with a Dunn's *post-hoc* test for multiple comparisons. Offspring health status was analyzed using Chi-Square test. Lung viral titers, progesterone and PGF2α concentrations, COX-2 and PIBF concentrations were analyzed using Two-way ANOVA using Sidak correction for multiple comparisons. Lung, placenta, and serum cytokine quantitation was analyzed via Two-way ANOVA and *post-hoc* multiple *T*-tests without assuming consistent SD with correction for multiple comparisons by controlling the false discovery rate per the two-stage set up method of Benjamini Krieger and Yekutieli (Q = 5%). Placental progesterone and PGF2α data were analyzed using Welch's *t*-test. Placental western blot data was analyzed using two-way ANOVA using Sidak correction for multiple comparisons. Cell frequencies in lungs, uterus, and placenta were analyzed via two-way ANOVA using Sidak correction for multiple comparisons. Antibody and cytokine secreting cell data were analyzed with two-way ANOVA using Sidak correction for multiple comparisons. All error bars represent Standard Error of Mean (SEM) and data represents the mean except Figures 9G,H, which are geometric means with gSD. Further experimental statistical details are described in the figure legends.

## Results

### Pandemic Influenza Infection Mid-pregnancy Attenuates Maternal Weight Gain and Shortens Gestation

To determine the impact of pandemic influenza virus infection on maternal health, we infected non-pregnant and E10-12 pregnant BALB/c mice with a low (0.5 × LD_50_) and a high (2 × LD_50_) dose of mouse adapted H1N1 A/California/07/2009 to recapitulate human infection at the end of 2nd to 3rd trimester of pregnancy.

**Figure 1:**
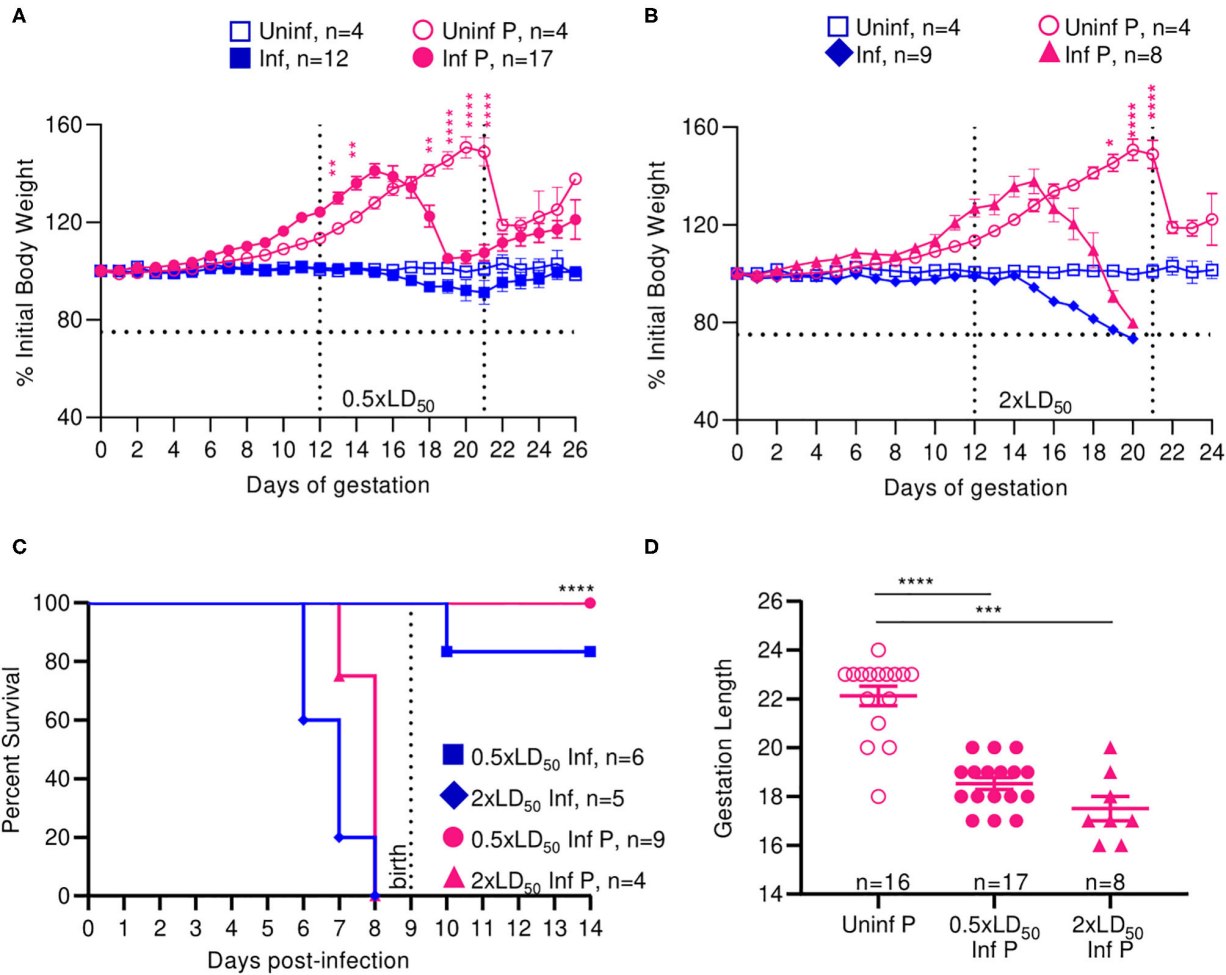
Infection with A/California/07/2009 attenuates maternal weight gain and reduces gestation length. Body weight changes across gestation were tracked and weight changes relative to weight at day 0 are plotted. Pregnant and non-pregnant mice were infected with either **(A)** 0.5- or **(B)** 2xLD50 of A/California/07/2009. Animals were infected at E12, denoted by the left-most vertical line. The horizontal line denotes the terminal weight endpoint. The right-most vertical line denotes expected delivery day following uncomplicated pregnancy **(C)** Percent survival curves comparing pregnant and non-pregnant animals infected with either 0.5- or 2xLD50. **(D)** Gestation length following infection with either 0.5- or 2xLD50. Each data point represents a single dam's delivery date. Error bars represent Standard Error of Mean (SEM) for all graphs. Weights were analyzed using Two-way ANOVA with Tukey post-host corrections. Survival data was analyzed using Mantel-Cox test and gestation length data was analyzed using a Kruskal-Wallis test with a Dunn's *post-hoc* test for multiple comparisons. *p < 0.05; **p < 0.01; ***p < 0.001; ****p < 0.0001.

Uninfected pregnant mice reached a peak bodyweight that was 50.67% greater than that of their pre-pregnancy bodyweight at 21 days of gestation (Figures 1A,B). Low dose (0.5 × LD_50_) infected pregnant animals reached a peak bodyweight of 41.2% greater than their original bodyweight at 16 days of gestation (Figure 1A), while the high dose (2 × LD_50_) infected pregnant animals reached a peak bodyweight of 37.8% greater than their original bodyweight at the same time point (Figure 1B). Following delivery, low dose infected pregnant mice lost 10% of their peak pregnancy weight through 26 days of monitoring, which was indistinguishable to the loss seen among uninfected pregnant controls. High dose influenza infected pregnant mice, however, lost 20% of their initial body weight following pre-term delivery, reaching moribund endpoints; this loss was comparable to the high dose infected non-pregnant controls, which were at 73.3% initial body weight by 6 days post-infection (DPI). These data demonstrated that both pregnant and non-pregnant cohorts had the same weight reduction following infection.

Median survival for high dose non-pregnant controls was 7 DPI and was insignificantly increased to 8 days for high dose infected pregnant mice. All mice infected with low dose survived through the 26-day monitoring period (*p* < 0.0001) (Figure 1C). While the average gestation period of uninfected pregnant controls was 22 days, it decreased by 19.4% to 18.5 days following low dose influenza infection (*p* < 0.0001), and 26.4% to 17.5 days following high dose influenza infection (*p* = 0.0001) (Figure 1D).

Infection with pandemic influenza H1N1 A/California/07/2009 virus interrupted the normal progression of pregnancy; we observed attenuated weight gain and pre-term labor in a dose dependent manner at both high and low infectious doses, and continued weight loss following delivery at high doses in our mouse model. Interestingly, we did not observe significantly differing responses in high dose infected pregnant and non-pregnant animals, as both these groups experienced comparable weight loss and complete mortality by day 8. Therefore, low dose infection recapitulates the clinical phenotype of morbidity and mortality observed in human women infected mid-gestation with pandemic influenza virus (2).

### Mice Born From Influenza Virus-Infected Mothers Have an Increased Likelihood of Adverse Outcomes

**Figure 2:**
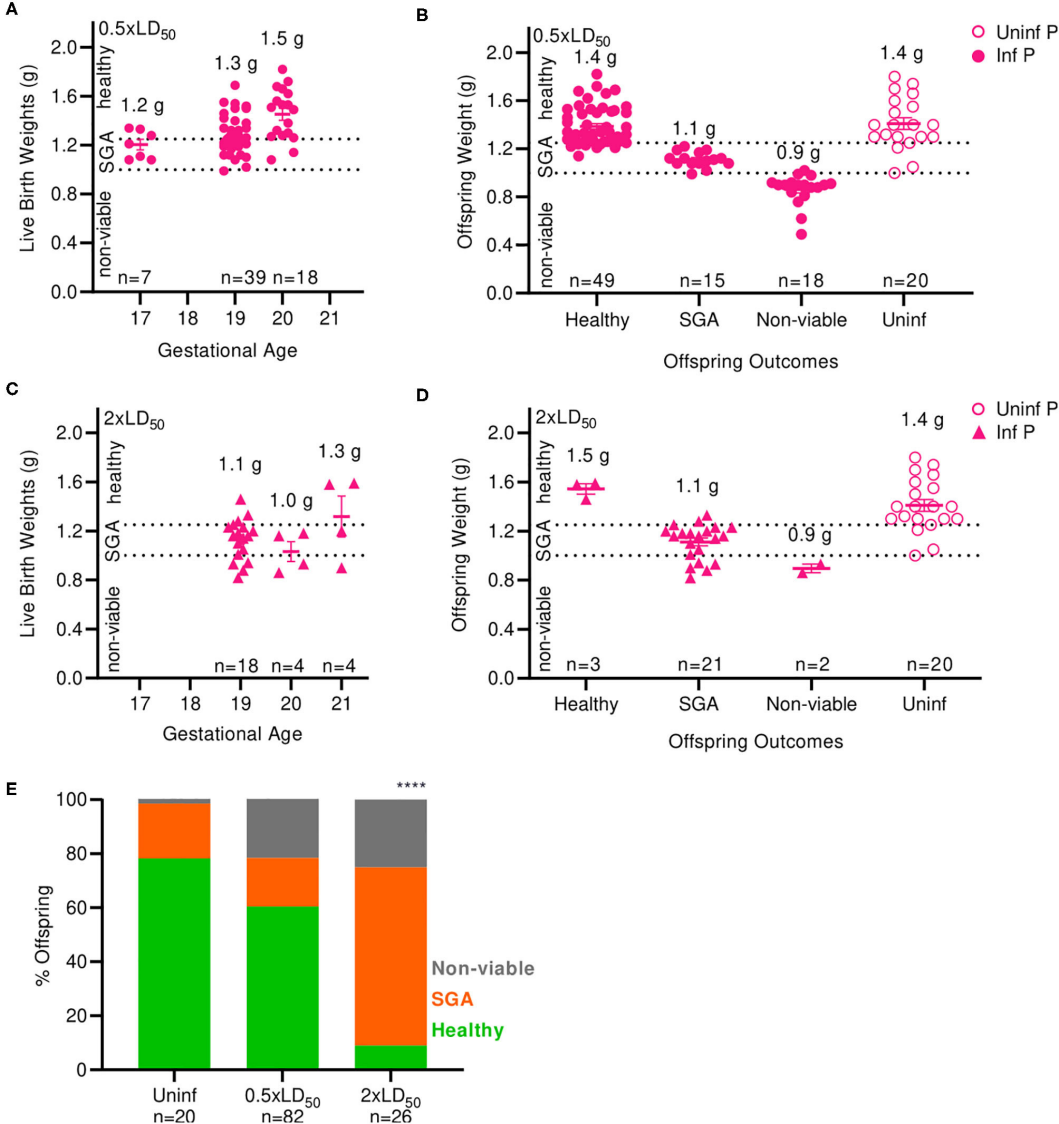
Maternal influenza infection inhibits offspring development and health. **(A)** Birth weights of live offspring were recorded from dams infected with 0.5xLD50. Numbers denoted above data clusters represent the average. **(B)** Offspring weights from 0.5xLD50 infections were recorded at the time of birth, including stillborn or live pups. **(C)** Birth weights of live offspring were recorded from dams infected with 2xLD50. Numbers denoted above data clusters represent the average. **(D)** Offspring weights from 2xLD50 infections were recorded at the time of birth, including stillborn or live pups. **(E)** Offspring health status at time of birth is plot as a percentage of all pups from mothers of either 0.5- or 2xLD50 infections. Numbers denoted above data clusters represent the average. Error bars represent Standard Error of Mean (SEM) for all graphs. Offspring health status was analyzed using Chi-Square test. ****p < 0.0001.

Next, we examined the impact of A/California/07/2009 infection on offspring viability and health status. Numbers of viable and non-viable pups were recorded; bodyweights were taken at birth and classified as non-viable (≤1.0 g), small for gestational age (SGA) (1.0–1.25 g) and healthy (>1.25 g). The average offspring weight from uninfected pregnant mice was 1.4 g (Figures 2B,D). Pups born from low dose infected mothers at 17 and 19 days of gestation had lower body weight compared to the pups born at day 20; average weight of live pups increased with length of gestation; 1.2 g at 17 days, 1.3 g at 19 days, and 1.5 g at 20 days (Figure 2A). When mothers were infected with a low dose of virus, the body weights of newborns were 15% lower than uninfected mothers, averaging 1.2 g (*p* = 0.0035) (Figure 2B). Similarly to the findings with low infectious dose, pups born from high dose infected mothers at 19 and 20 days of gestation had lower body weight compared to the pups born day 21; average weight of live pups were 1.1 g at 19 days, 1.0 g, 20 days, and 1.3 g 21 days (Figure 2C). When mothers were infected with a high dose of virus, the body weight of newborns was 23% lower than uninfected controls, averaging 1.14 g (*p* = 0.0001) (Figure 2D). In uninfected pregnant mothers, 78.3% of pups were born with a healthy weight, while 20.3% were SGA and 1.4% were stillborn (Figure 2E). Infection with low dose pandemic influenza virus reduced the frequency of healthy offspring to 60.4%; the remaining pups were 18% SGA, and 22% stillborn. Infection with high dose pandemic influenza virus dramatically reduced the frequency of healthy offspring to 9%; the overwhelming majority of pups were SGA (66%), and 25% were stillborn (*p* < 0.0001).

Overall, offspring born from infected mothers had reduced body weight compared to offspring of uninfected mothers. A low dose of influenza greatly increased the incidence of stillborn pups from 1.4 to 22%; a high dose further increased the percentage of stillbirth to 25% of offspring, an 18-fold increase over uninfected dams. The frequency of SGA offspring increased by 3.3-fold at high infectious dose compared to uninfected control mothers. Low birthweight is a common outcome of pregnant mothers infected with pandemic H1N1 influenza virus during the second and third trimester (2); thus, our model replicates this serious clinical pregnancy complication associated with influenza A infection during mid- to late-gestation pregnancy.

### Pregnancy Increases Lung Viral Titers and Pathology in Influenza Infected Mice

**Figure 3:**
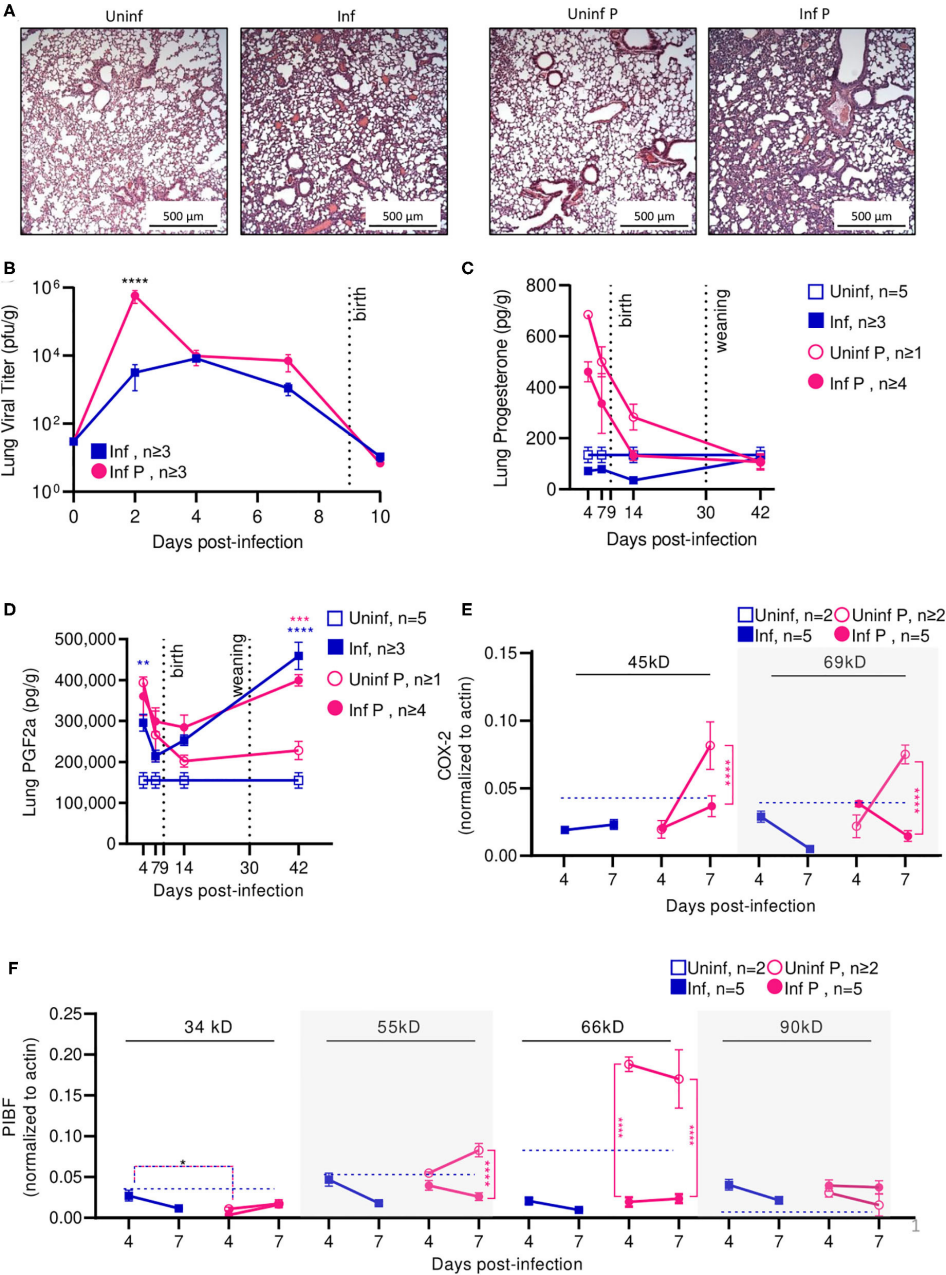
Pregnant mice demonstrate elevated viral titers in lungs and show dysregulated hormone-mediated inflammatory responses during influenza infection. **(A)** Representative H&E histology of lungs from pregnant and non-pregnant mice infected with 0.5xLD50 at 4 DPI. **(B)** Viral titer was determined for pregnant and non-pregnant infected animals by plaque-forming assay at 0, 2, 4, 7, and 10 DPI. Concentrations of **(C)** progesterone and **(D)** PGF2α from lung protein lysates were analyzed at 4, 7, 14, and 42 DPI. **(E)** Western blots on **(E)** COX-2 and **(F)** PIBF protein concentrations from lung lysates at 4 and 7 DPI were normalized to β-actin. Unglycosylated isomers of COX-2 were detected at 45 kD, while glycosylated forms were observed at 69 kD. PIBF isoforms were observed at 34, 55, 66, and 90 kD. Blue dashed horizontal lines indicate the average value of uninfected non-pregnant control animals. Error bars represent Standard Error of Mean (SEM) for all graphs. All graphs were analyzed using Two-way ANOVA using Sidak correction for multiple comparisons. *p < 0.05; **p < 0.01; ***p < 0.001; ****p < 0.0001.

To evaluate the time course of A/California/07/2009 infection during mid-gestation, we further examined our low dose pregnant mouse model due to higher survival prognosis. The gravity of influenza infection was assessed by histological examination of lungs at 4 DPI (Figure 3A). Both non-pregnant and pregnant infected lungs showed evidence of neutrophilic infiltration, occluded blood vessels, and goblet cell hyperplasia resulting in thick mucus lining the bronchoalveolar spaces; however, pregnancy exacerbated leukocytic infiltration and goblet cell hyperplasia. The worsened pulmonary pathology in pregnant infected mice coincided with 180-fold higher viral loads as measured by plaque forming units (*p* < 0.0001) in pregnant animals compared to non-pregnant at 2 DPI (Figure 3B), suggesting uncontrolled viral replication precipitating disease.

Progesterone is vital to fetal-placental development and increases during gestation are associated with relaxation of maternal airway smooth muscle and bronchial tissue dilation (55). In the absence of infection, lung progesterone of pregnant animals showed a decreasing trend from days 4 to 14. When compared to non-pregnant controls, pregnant animal lung progesterone was 5.1- and 3.7-fold higher at 4 DPI and 7 DPI (*p* < 0.0001), respectively; these groups became statistically indistinguishable 12 days after weaning (Figure 3C). Following sub-lethal infection, pregnant lungs demonstrated a 50% reduction of lung progesterone up to 14 DPI compared to uninfected pregnant controls.

PGF2α is a naturally occurring prostaglandin which is clinically used to induce labor or as an abortifacient (56) and acts in opposition to the effects of progesterone. A key player in lung response to infection, PGF2α causes bronchoconstriction (57). Infection increased PGF2α in non-pregnant mice compared to uninfected controls by 2-fold 4 DPI (*p* = 0.003) (Figure 3D). In the context of pregnancy, PGF2α opposes progesterone-associated bronchodilation to finely tune lung physiological function (57). High PGF2α levels before the onset of labor have been previously reported (58). While uninfected pregnant mice had 2.5-fold higher PGF2α compared to uninfected non-pregnant controls (*p* = 0.004), this difference disappeared following delivery (*p* = 0.78 14 DPI). Contrary to the pronounced PGF2α response seen in non-pregnant mice, infection did not increase PGF2α levels in pregnant mice. However, pregnant infected and non-pregnant infected controls had elevated lung PGF2α through 42 DPI. Infected pregnant mice had 1.8-fold higher (*p* = 0.0002), and infected non-pregnant 3-fold higher (*p* < 0.0001) PGF2α than their respective uninfected controls. Therefore, while PGF2α levels were maintained following influenza infection, reduced progesterone levels and the negative feedback loop between PGF2α:progesterone suggest a mechanism for reduced gestation length.

We further evaluated hormone regulators cyclooxeganse-2 (COX-2) and progesterone-induced blocking factor (PIBF). COX-2 is a key regulator in the arachidonic acid and prostaglandin synthesis pathway, and increased COX-2 expression is associated with increased PGF2α secretion by bronchial epithelial cells (59). Reductions in COX-2 expression have been associated with decreased macrophage and neutrophil recruitment to sites of influenza infection, resulting in impaired virus clearance (60). Our results point to an infection-specific inhibition of post-translational modification for both pregnant and non-pregnant infected groups. In non-pregnant infected animals, the glycosylated (functional) 69 kD isoform of COX-2 was 4.9-fold lower than the non-glycosylated (non-functional) 45 kD isoform of COX-2 at 7 DPI (*p* = 0.01) (Figure 3E). In pregnant infected animals, COX-2 69 kD was 5.3-fold lower than COX-2 45 kD at the same timepoint (*p* = 0.01). This differential concentration of COX-2 isoforms was not observed in either uninfected animals. Further, the 45 kD COX-2 isoform was decreased 2.2-fold in lungs of infected pregnant mice compared to uninfected pregnant controls (*p* < 0.0001), and the 69 kD form was reduced 5.3-fold (*p* < 0.0001). This reduction was not significant in non-pregnant infected animals.

Successful pregnancies require expression of PIBF, which promotes Th2 cytokine production and inhibition of NK cell activity, resulting in reduced cytolytic activity at the fetal-maternal interface and potentially increases maternal susceptibility to infection (25). This progesterone-responsive immunomodulatory protein has multiple splice variants. The nuclear-associated 90 kD isoform has been implicated in control of embryotic cell differentiation, development, and/or stem cell maintenance in the developing fetus via control of mitotic activity; smaller 66, 55, and 34 kD variants potentially act as both transcription factors and cytokines, with the 34 kD isoform specifically inhibiting NK cell function while simultaneously inducing their homing to the placenta (61). In the absence of infection, mice late in gestation (E16) demonstrated 3-fold lower isoform 34 kD, whereas isoforms 66 and 90 kD were 2- and 6-fold higher when compared to non-pregnant mice (Figure 3F). The 34 kD isoform was differentially regulated in pregnant and non-pregnant animals; at 4 DPI, the non-pregnant infected animals had 9.1-fold higher PIBF 34 kD than infected pregnant animals (*p* = 0.03). However, infection did not significantly affect PIBF 34 kD in pregnant animals (*p* > 0.05 for 4 and 7 DPI). The 55 kD isoform was differentially affected by infection only in pregnant mice; at 7 DPI, uninfected pregnant mice had 3.2-fold higher levels of this protein in the lungs than infected pregnant mice (*p* < 0.0001). The 66 kD splice variant was strongly downregulated in pregnant infected mice compared to uninfected pregnant controls; at 4 DPI, uninfected mice had 9.7-fold higher expression (*p* < 0.0001), and at 7 DPI, uninfected mice had 7.2-fold higher expression (*p* < 0.0001). This loss of immunomodulatory PIBF 55 and 66 kD during infection may explain the reduced gestation length in mice infected with 0.5 × LD_50_ A/California/07/2009 due to reduced inhibition of NK cell activity. Lastly, the 90 kD isoform of PIBF was unaffected by infection or pregnancy. The lack of pregnancy-associated changes in PIBF isoforms 34 and 90 kD in the lungs suggests that these isoforms may exert a maternal protective mechanism.

**Figure 4:**
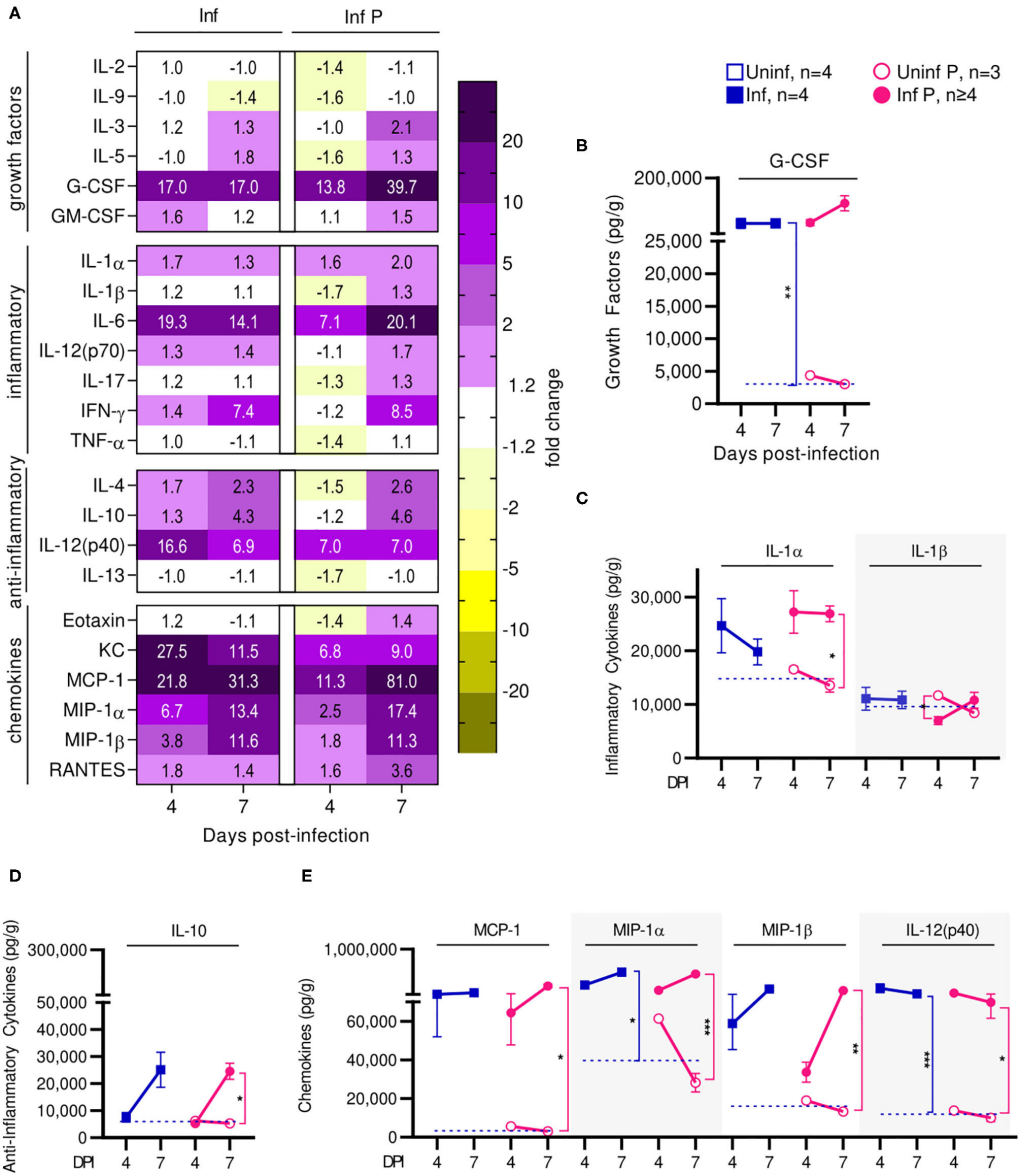
Pregnant females show delayed lung inflammation following influenza infection. **(A)** Lung protein concentrations from infected non-pregnant and pregnant mice from 4 and 7 DPI were quantified for growth factors, inflammatory and anti-inflammatory cytokines and chemokines. Values within the heat-map denote mean fold change over uninfected controls. Fold change was transformed as follows to aid in visualization of decreases: if fold change >1, no transformation; if fold change <1, –(10 | log10fold change |). Concentrations of the growth factor **(B)** G-CSF, inflammatory cytokines **(C)** IL-1α and IL-1β, and regulatory cytokine **(D)** IL-10 were quantified. **(E)** Chemokines MCP-1, MIP-1α, MIP-1β, and IL-12(p40) were also quantified at 4 and 7 DPI. Cytokine quantitation was analyzed via Two-way ANOVA and *post-hoc* multiple T-tests without assuming consistent SD with correction for multiple comparisons by controlling the false discovery rate per the two-stage set up method of Benjamini Krieger and Yekutieli (Q = 5%). Blue dashed horizontal lines indicate the average value of uninfected non-pregnant control animals. *p < 0.05; **p < 0.01; ***p < 0.001.

Infection affected recruitment of innate immune populations to the lungs of pregnant mice compared to non-pregnant controls. At 4 DPI (Figure 4A, Supplementary Table 1), we found upregulation of G-CSF by 17-fold, KC by 27.5-fold, MIP-1α by 6.7-fold, and RANTES by 1.8-fold in infected non-pregnant mice (Figure 4D). At the same time point, pregnant mice upregulated a number of inflammatory chemokines and cytokines: G-CSF by 13.8-fold, KC by 6.8-fold, MCP-1 by 11.3-fold, MIP-1α by 2.5-fold, RANTES by 1.6-fold, IL-12p40 by 7-fold, and IL-6 by 7.1-fold. However, IL-1β was downregulated 1.7-fold (*q* = 0.04) (Figure 4C). When comparing pregnant infected mice to non-pregnant infected controls, we observed a downregulation of TH1, TH2 and Treg cytokines. Infection in pregnant mice induced a 1.6-fold reduction of KC, 1.7-fold reduction in MIP-1α, 1.4-fold reduction of RANTES, 2.4-fold reduction of IL-6 and 2-fold reduction of IL-12(p40) at 4 DPI (Figure 4E), compared to non-pregnant controls.

At 7 DPI, pregnant infected mice had a 39.7-fold increases in G-CSF, while in non-pregnant mice, G-CSF increased 17-fold (*q* < 0.01) (Figure 4B). Pregnant infected mice also had a 2-fold increase of IL-1α in the lungs, compared to pregnant uninfected (*q* = 0.02) (Figure 4C). Infected pregnant mice also exhibited a 4.6-fold increase in IL-10 compare to uninfected controls (*q* = 0.04) (Figure 4D). Lastly, both infected pregnant and non-pregnant groups maintained increases of KC, MCP-1, MIP-1α, MIP-1β, RANTES, IL-14(p40), and IL-6, reaching similar levels in both cohorts with the exception of MCP-1 which was increased 81-fold (*q* = 0.03) in infected pregnant mice (Figures 4A,E, Supplementary Table 2).

Thus, preterm birth in our model is correlated with increased acute viral titers and enhanced lung pathology. Cytokine profiling suggests that the status of pregnancy contributes to acute lung inflammation through immune mediators that affect recruitment of innate and adaptive immune cells to the lungs at peak influenza viremia. We observed upregulated mediators of monocytes and neutrophil recruitment at the lungs, which may account for increased acute viral titers and enhanced pathology. Further, infection resulted in long term PGF2α upregulation which is responsible for bronchoconstriction and pulmonary vasoconstriction leading to reduced vital capacity, respiratory compromise, and retardation of fetal growth. Lung progesterone—a bronchodilator that increases with pregnancy—factors in respiratory distress since its reduction leads to bronchoconstriction (55). Furthermore, downregulation of PIBF isoforms which support pregnancy following infection correlates with threatened pregnancy. Finally, post-transcriptional protein modification seems to be inhibited in infected animals independent of pregnancy status, as evinced by COX-2 which demonstrated reduced glycosylation ratios.

### At Peak Pathogenicity, Pregnancy Exacerbates Infection-Response-Induced Serum Cytokine Levels

Placental or uterine inflammation caused by infection or autoimmune responses may induce adverse pregnancy outcomes, including preeclampsia, endometriosis, and spontaneous abortion (62). However, it has not been demonstrated that influenza virus can cross the maternal-fetal barrier in mice or humans, and previous studies in our group have examined placenta from infected mice and found no evidence of direct viral infection (12). Therefore, we next evaluated influenza-induced alterations to blood hormones and immune mediators as a connection between the site of influenza infection and local, placental perturbations.

Serum progesterone was unaffected by infection in the non-pregnant cohort (*p* > 0.5 at all timepoints) but was increased 2.3-fold in the infected pregnant animals compared to the uninfected pregnant controls 4 DPI (*p* = 0.0004) (Supplementary Figure 1A). Serum PGF2α was lower in all groups compared to values detected in the lungs. While none of the timepoints reached statistical significance, we observed a trend of peak PGF2α levels in serum at 7 DPI in pregnant animals that was not evident in non-pregnant mice (Supplementary Figure 1B). Additionally, there was a tendency for infected animals to have reduced PGF2α levels long after viral resolution; infected pregnant animals had 5.4-fold lower PGF2α 42 DPI than the uninfected pregnant mice (*p* = 0.09).

Cytokine production analysis revealed that infected non-pregnant and pregnant animals had similar changes respective to their uninfected controls at 4 DPI (Supplementary Table 3, Supplementary Figure 1C). We observed temporarily reduced production of most immune mediators for both cohorts. By 7 DPI (Supplementary Table 4, Supplementary Figure 1C), infected non-pregnant animals demonstrated modest upregulation of inflammatory cytokines IL-1β, IL-6, eotaxin, MCP-1, and MIP-1β compared to uninfected controls. Infected pregnant mice, on the other hand, had substantial upregulation of inflammatory cytokines with higher concentrations of IL-1β (4.4-fold increase), MCP-1 (3.1-fold increase), MIP-1α (1.8-fold increase), TNF-α (2.3-fold increase), and IL-6 (2.5-fold increase) compared to uninfected non-pregnant mice. IL-17 production was 2-fold higher in pregnant animals prior to infection compared to non-pregnant animals. Infected pregnant mice had 1.9-fold higher TH1 cytokine IFN-γ and 4.4-fold higher T-cell growth factor IL-9 uninfected controls.

In sum, patterns of cytokine production in serum in response to influenza infection were similar between pregnant and non-pregnant animals; however, at 7 DPI, pregnant animals tended to have exacerbated cytokine and chemokine production compared to non-pregnant infected controls. This presence of inflammatory cytokines and chemokines in the serum may play a role in adverse outcomes at the fetal-maternal interface.

### Influenza **Infection** Abrogates Pregnancy-Supporting Hormone Levels and Destroys Placental Architecture Through Local Granulocytes Recruitment and Activation

**Figure 5:**
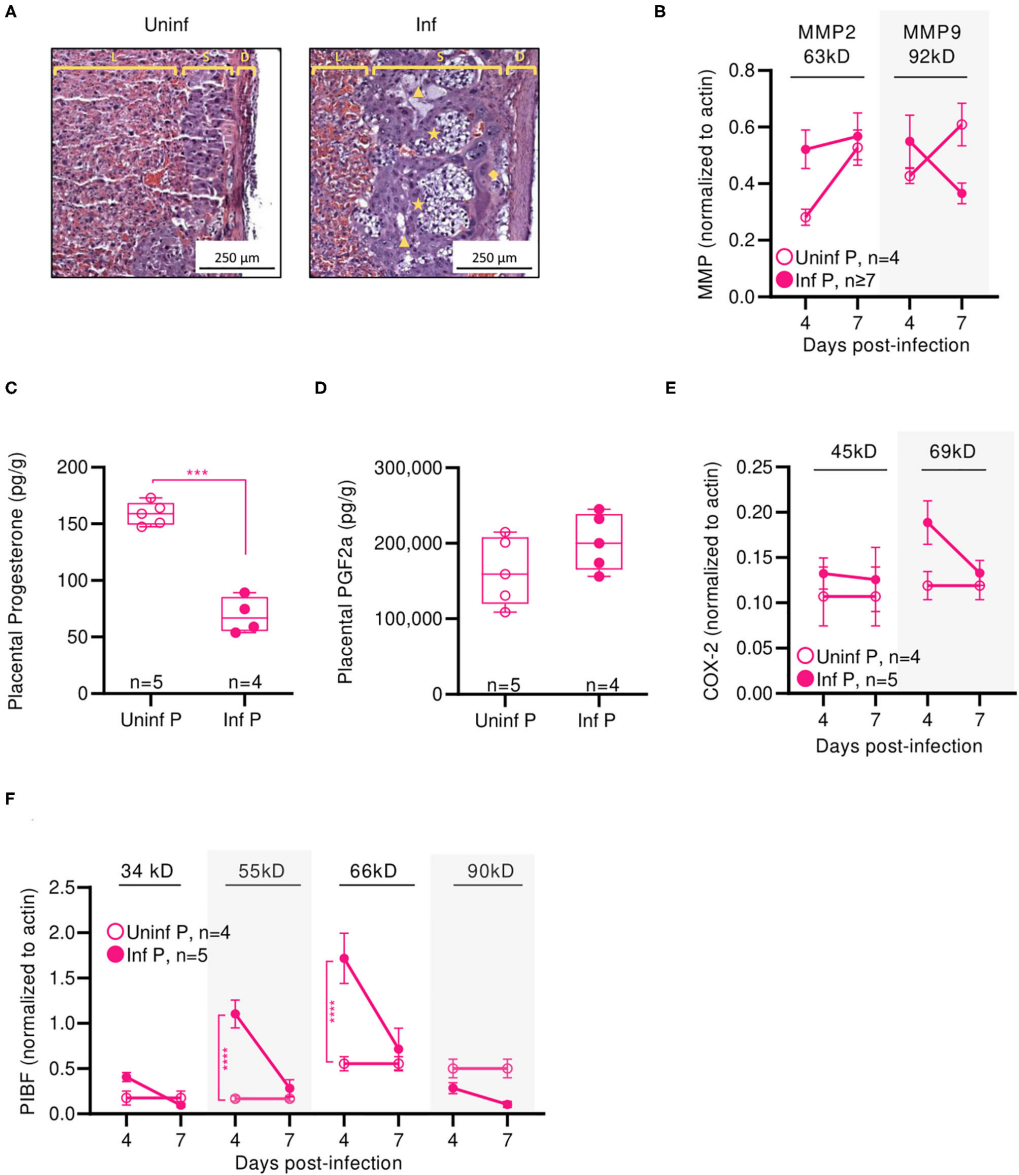
Influenza infection induces short-term placental inflammation and subsequent vascular remodeling. **(A)** Representative H&E histology of placenta from mice infected with 0.5xLD50 or uninfected at 4 DPI (E14-16): spongiotrophoblast layer (S) between the decidual layer (D) and the placental labyrinth (L) with fetal cell hyperplasia [★] and empty space [▲] distinct from maternal blood sinuses and fetal blood vessels and fibrinoid necrosis [♦] beneath the decidual layer. Western blots for **(B)** MMP2 and MMP9 proteins were run on at 4 and 7 DPI. Concentrations of progesterone **(C)** and PGF2α **(D)** were determined in placental protein lysates at 4 DPI. Western blots for **(E)** COX-2 isoforms and **(F)** PIBF isoforms were run on at 4 and 7 DPI. COX-2 isoforms were observed at 45 and 69 kD. PIBF isoforms were observed at 34, 55, 66, and 90 kD. All Western bots were normalized to β-actin. Progesterone and PGF2α data were analyzed using Welch's *t*-test. Western blot data was analyzed using Two-way ANOVA using Sidak correction for multiple comparisons. ***p < 0.001; ****p < 0.0001.

We have previously determined that H1N1 influenza viral titers and viral RNA are undetectable in the placenta or fetuses of infected mice, suggesting that vertical transmission of influenza virus is unlikely and adverse events such as shortened gestation time, stillbirth, or SGA offspring are not the result of *in utero* infection (12). This implies that adverse outcomes on fetal health are brought about by indirect causes such as dysregulated hormone signaling, excess inflammation, or loss of placental function. As the placenta is the sole source of nutrients and oxygen to the fetus, we histologically evaluated placental architecture and function at E16, 4 DPI (Figure 5A). Placentae from uninfected dams showed structural integrity between the maternal decidual layer and the fetal placental spongiotrophoblast and labyrinth layers. Placentae from infected mothers, on the other hand, demonstrated significant loss of architecture, particularly between the maternal and fetal layers, suggesting poor placental health. The decidua of infected mice was hyperplastic. Further, we observed regions of fetal cell hyperplasia (★), increased gaps within the spongiotrophoblast layer (S; ▲), and increased fibrinoid necrosis at the intersection of fetal and maternal layers (◆). To further evaluate the molecular causes of placental disorganization and adverse pregnancy outcomes, we characterized placental expression of matrix metalloproteinases (MMPs) in the absence or presence of influenza infection. For both bioactive MMP-2 and MMP-9, we observed 1.9- and 1.3-fold increases early after infection (4 DPI), suggesting that both the placenta and the surrounding fetal membrane were subjected to enhanced MMP-mediated degradation as a result of influenza infection (Figure 5B). These differences disappeared by 7 DPI for MMP-2. For MMP-9, uninfected pregnant had 1.7-fold higher concentrations, potentially as preparation for labor and delivery (62).

We next determined if loss of placental integrity was accompanied by reduction in pregnancy-supportive progesterone. Indeed, infected pregnant mice had 2.9-fold less progesterone in placental lysates than uninfected pregnant controls (*p* = 0.0002) (Figure 5C). As progesterone is partially responsible in suppressing inflammation in the uterus, we measured infection-induced changes in PGF2α which can stimulate the production of pro-inflammatory cytokines. These may result in neutrophil activation (63) or labor-inducing uterine blood vessel vasoconstriction (64). While the difference did not reach statistical significance, we saw a trend of infected placentae having 1.2-fold more PGF2α than uninfected controls (Figure 5D). PGF2α-regulating COX-2 45 kD was unaffected by infection whereas the 69 kD isoform was reduced 50% at 4 DPI (Figure 5E). Expression of progesterone-responsive immunomodulatory protein PIBF isoforms echoed placental progesterone patterns: while the 34 and 90 kD isoforms were unchanged between infected and uninfected pregnant animals, expression of the 55 and 66 kD were 6.6-fold (*p* < 0.0001) and 3.1-fold (*p* < 0.0001) lower, respectively, in infected animals than uninfected controls 4 DPI (Figure 5F). Thus, adverse pregnancy outcomes in our model were correlated with loss of placental architecture and infection-associated reductions in pregnancy-supportive progesterone and PIBF.

**Figure 6:**
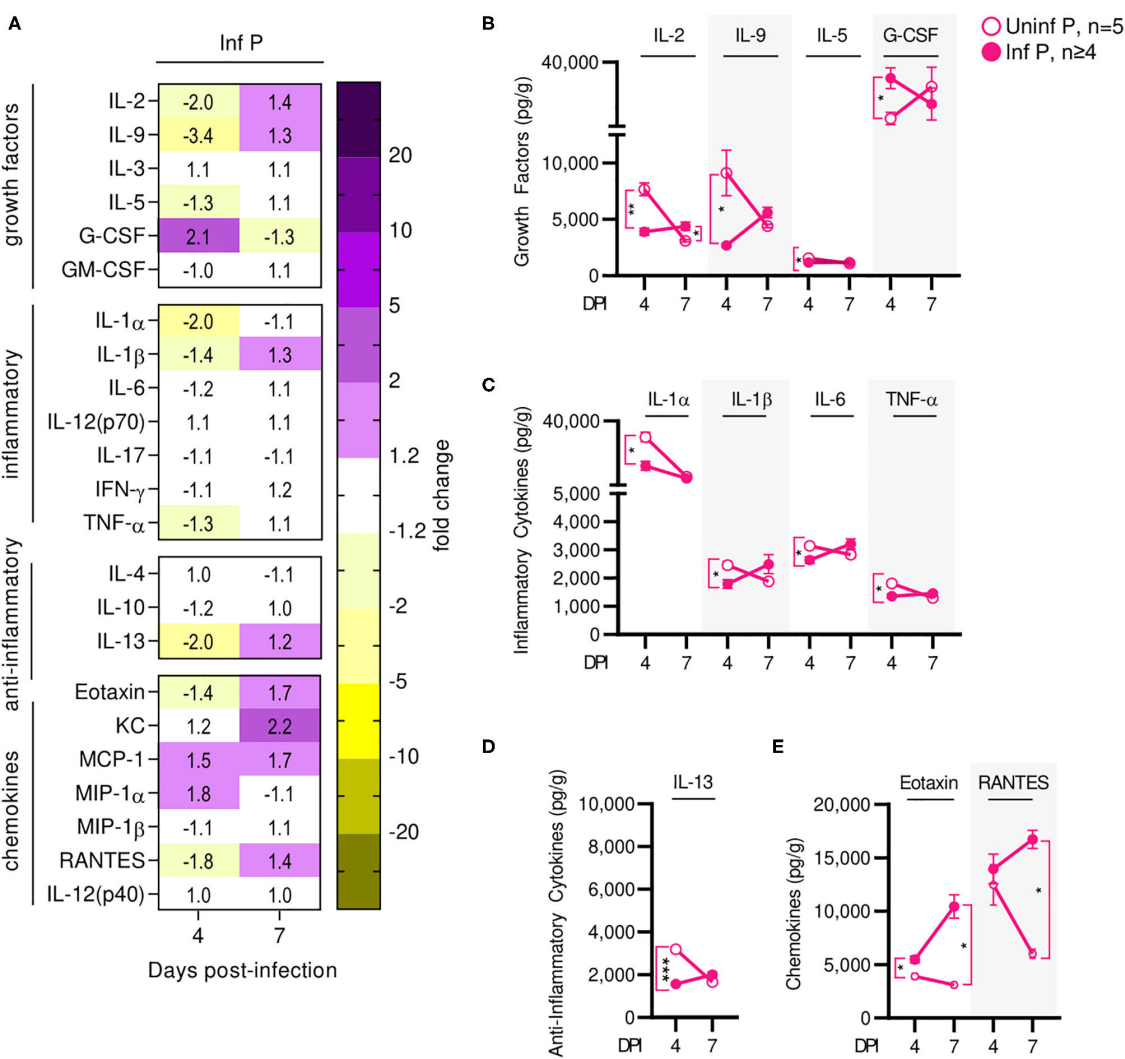
Influenza infection results in an early increase of placental macrophage-secreted T cell stimuli. **(A)** Protein concentrations from infected placental lysates collected at 4 and 7 DPI were normalized to uninfected controls. Lysates were quantified for growth factor, inflammatory and anti-inflammatory cytokine, and chemokine concentrations. Values within the heat-map denote mean fold change over uninfected controls. Fold change was transformed as follows: if fold change >1, no transformation; if fold change <1, –(10 | log10fold change |). Concentrations of growth factors **(B)** IL-2, IL-9, IL-5, and G-CSF, the inflammatory cytokines **(C)** IL-1α, IL-1β, IL-6, and TNF-α, anti-inflammatory cytokines **(D)** IL-13, and the chemokines **(E)** eotaxin and RANTES were quantified at 4 or 7 DPI. Cytokine quantitation was analyzed via Two-way ANOVA and *post-hoc* multiple *T*-tests without assuming consistent SD with correction for multiple comparisons by controlling the false discovery rate per the two-stage set up method of Benjamini Krieger and Yekutieli (Q = 5%). *p < 0.05; **p < 0.01; ***p < 0.001.

Influenza infection-induced changes in cytokine production at the placenta of infected mice were measured and compared to uninfected pregnant controls. Similarly to serum cytokine patterns, global placental cytokine levels were depressed compared to uninfected controls at 4 DPI (Figure 6A, Supplementary Table 5). At this time point, we observed downregulation of IL-1α by 2-fold (*q* = 0.03), IL-1β 1.4-fold (*q* = 0.03), IL-6 1.2-fold (*q* = 0.03), and TNF-α by 1.3-fold (*q* = 0.03) (Figure 6C) and a 2-fold reduction in IL-13 (*q* < 0.01) (Figure 6D). We also observed a 1.4-fold reduction in eotaxin (*q* = 0.03) (Figure 6E), and down regulation of growth factors IL-2 (by 2-fold, *q* < 0.01), IL-9 (by 3.4-fold (*q* = 0.04), and IL-5 (by 1.3-fold, *q* = 0.03) (Figure 6B). However, levels of prostaglandin-independent fever-inducer MIP-1α were 1.8-fold higher in infected pregnant mice. MIP-1α acts to attract granulocytes which could be stimulated to survive, proliferate, differentiate, and function by 2-fold increased GM-CSF observed in pregnant mice (*q* = 0.03) (Figure 6B) in placentas of infected dams. By 7 DPI, no changes were observed in the cytokine/chemokine panel with the exception of 1.4-fold increased of IL-2 (*q* = 0.03) (Figure 6B). Additionally, infection-associated neutrophil chemoattractant KC concentrations increased from 1.2- to 2.2-fold, eotaxin concentrations increased to 1.7-fold (*q* = 0.03), and RANTES concentration increased 1.4-fold above control (*q* = 0.03) (Figure 6E).

Overall, most cytokines were increased much less than their serum counterparts; the only cytokines increased specifically at the placenta were T-cell growth factors. This indicates that the placenta and the fetuses are mostly shielded from the maternal inflammatory cytokine signature. Recruitment and activation of granulocytes to the placenta by the local cytokine milieu may mediate observed loss of placental architecture and, in conjunction with loss of progesterone and PIBF, induce poor fetal outcomes.

### Influenza Alters Lung and Fetal-Maternal Interface Immune Cell Populations

**Figure 7:**
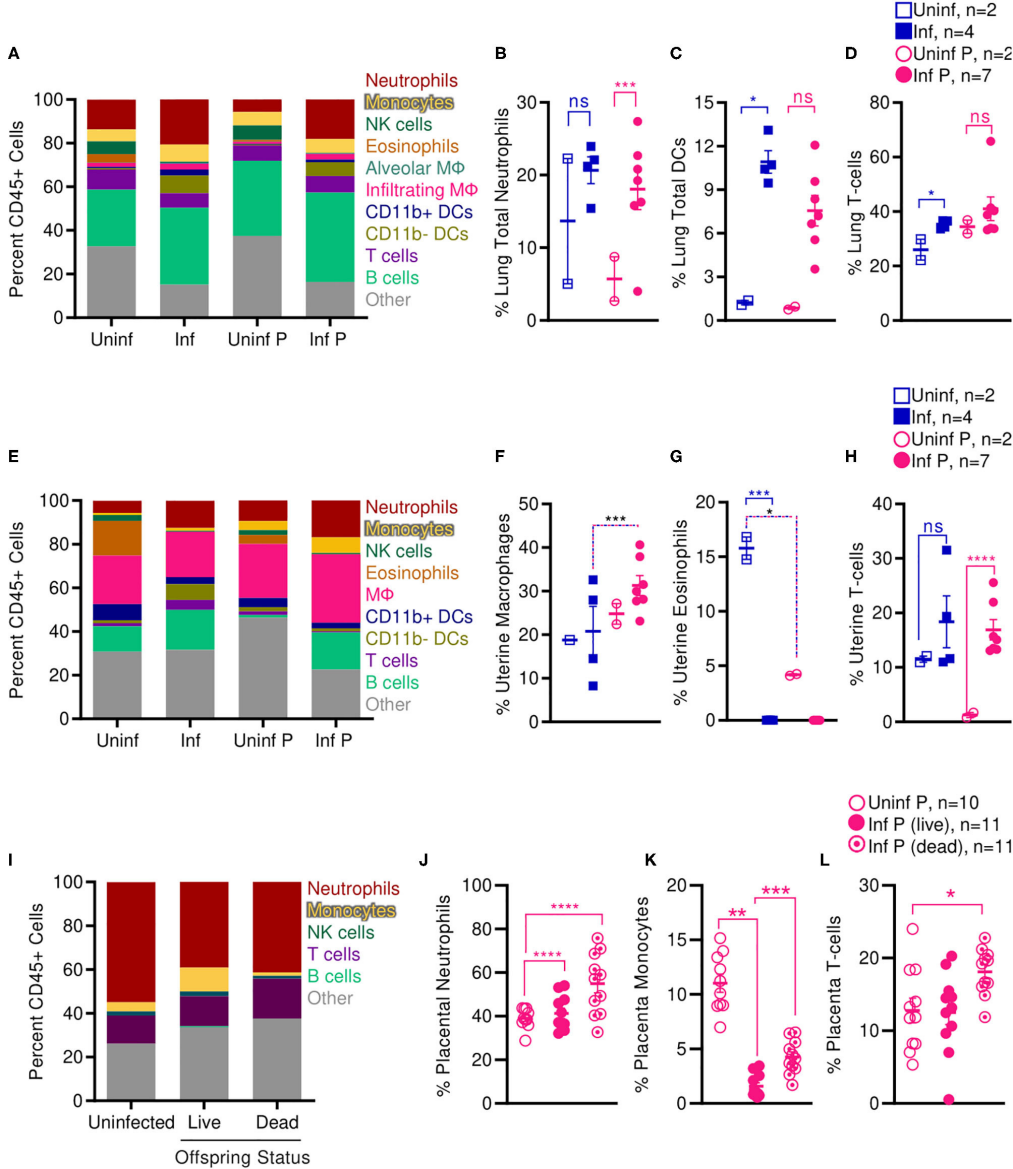
Pregnant mice show increased pulmonary T cells, uterine macrophages, and placental neutrophils following influenza infection. Immune cell populations were determined from total CD45+ cells by flow cytometry in **(A–D)** lungs, **(E–H)** uteruses, and **(I–L)** placenta isolated from infected and uninfected pregnant and non-pregnant animals. Cell frequencies were analyzed via Two-way ANOVA using Sidak correction for multiple comparisons. Error bars represent Standard Error of Mean (SEM) for all graphs. *p < 0.05; **p < 0.01; ***p < 0.001; ****p < 0.0001.

Increased activation of systemic innate immunity is a hallmark of pregnancy, and specific innate immune responses, including neutrophil and monocyte responses, are exacerbated (5, 65, 66). On the other hand, some aspects of adaptive immunity, such as T cell and B cell frequencies, have been reported to decrease during pregnancy (5, 65, 66). Therefore, we evaluated frequencies of innate and adaptive immune cells at the site of influenza virus infection E14, 2 DPI (Supplementary Figure 2). We found that infection during pregnancy significantly increased the proportion of neutrophils in the lungs from 5.7 to 18.0% (*p* = 0.0001) whereas infection did not affect the percent of lung neutrophils in non-pregnant animals (Figures 7A,B). However, we observed the opposite pattern in the adaptive immune responses: contrary to infected non-pregnant mice, which showed an increase in total lung DCs from 1.2 to 10.9% (*p* = 0.011) (Figures 7A,C) and lung T-cells from 26.0 to 35.3% (*p* = 0.016) (Figures 7A,D), pregnant infected animals did not have significant changes in either DC or T-cell populations as a result of infection.

While systemic innate immunity is considered to be generally activated in pregnancy, uterine innate immunity depends on age of gestation: early in pregnancy, innate immunity assists in implantation through an inflammatory response; during the second and third trimester, innate immunity plays a regulatory role; and finally, inflammation is renewed with parturition and labor (5). Therefore, we evaluated pregnancy and influenza induced changes to immune cell populations at the uterus and the placenta mid-gestation (2 DPI at E14). In the absence of infection, pregnant animals had 3.8-fold fewer uterine eosinophils than non-pregnant controls (*p* = 0.04); infection resulted in total loss of all uterine eosinophils for both non-pregnant (*p* < 0.001) and pregnant animals (Figures 7E,G). Pregnant infected animals had 1.5-fold more uterine macrophages than non-pregnant infected animals (*p* < 0.001) (Figures 7E,F). Lastly, in pregnant animals, infection increased the proportion of uterine T-cells from 1.3 to 16.9% (*p* < 0.0001) while this did not occur in non-pregnant animals (Figures 7E,H).

We next evaluated immune cell populations in the placenta of non-infected mice and compared them to the populations of cells found in the placenta of live and dead pups from influenza infected mothers. Infection significantly increased proportions of placental neutrophils independent of health status of the pups; while uninfected placenta had 39% neutrophils, infected placenta from live pups had 41.3% neutrophils (*p* < 0.0001), and placenta from dead pups had 54.9% neutrophils (*p* < 0.0001) (Figures 7I,J). This increase in neutrophils was countered by a dramatic loss of placental monocytes. Monocytes made up 11% of immune cells in placenta from uninfected animals, dropped to 1.6% in infected placenta that gave live pups (*p* = 0.009) and 4.3% in infected placenta from stillborn pups. Within the infected pregnant cohort, placentas from stillborn pups had 2.7-fold more monocytes than placentas from live pups (*p* = 0.0001) (Figures 7I,K). Lastly, placenta from stillborn pups had a 1.4-fold increase in placental T cells compared to placenta from uninfected mice (*p* = 0.0471) (Figures 7I,L).

Enhanced lung pathology in our pregnant mouse model was accompanied by increased neutrophilic proportions and reduced local adaptive immune populations. Shortened gestation times, stillbirth, and SGA pups following sublethal infection from influenza virus in our model were associated with increased macrophages and T cells in the uterus, and increased neutrophils and T cells in the placenta. Loss of placental monocytes following influenza infection may further propagate loss of placental architecture and fetal tolerance, as placental macrophages play pivotal roles in placental homeostasis and immunity.

### Pregnancy Confounds Cellular Adaptive Immune Responses

**Figure 8:**
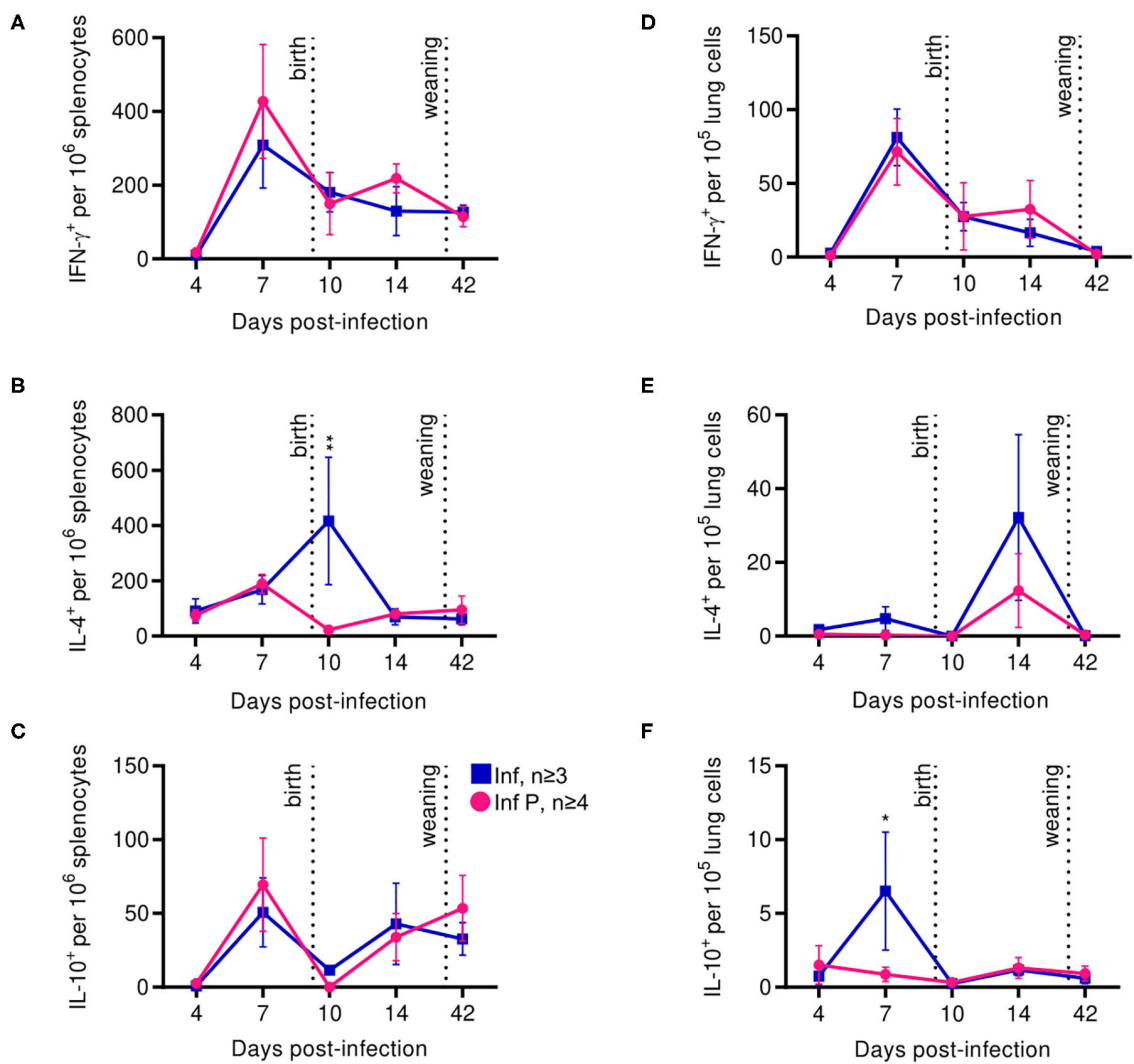
Proinflammatory T cell secretion profiles among pregnant mice are indistinguishable from non-pregnant animals, while anti-inflammatory secretions increase in the spleen after delivery. ASCs were quantified for IFN- γ **(A)**, IL-4 **(B)**, and IL-10 **(C)** secretion among splenocytes by ELISpot assay at 4, 7, 10, 14, and 42 DPI. IFN- γ **(D)**, IL-4 **(E)**, and IL-10 **(F)** secretion were also quantified in lung cells for the same days. All data was analyzed via Two-way ANOVA using Sidak correction for multiple comparisons. Error bars represent Standard Error of Mean (SEM) for all graphs. *p < 0.05; **p < 0.01.

In addition to some aspects of adaptive immunity becoming less frequent, functional capabilities of the adaptive immune system, such as the ability of CD4^+^ T cells to produce TH1– and TH2-type cytokines, have been reported to decrease during pregnancy (5, 65, 66). Therefore, we examined systemic and local T cell functions through interrogating cytokine production by influenza-specific cells in the spleen and lungs. In the spleen, kinetics of IFN-γ or IL-10 secreting T-cells after influenza infection were unaffected by pregnancy (Figures 8A,C). However, splenic IL-4 secreting T cells were 18.4-fold less frequent at in pregnant infected animals compared to non-pregnant mice at 10 DPI (*p* = 0.0045) (Figure 8B). Influenza infection-induced IFN-γ secreting cells in the lung were unaffected by pregnancy (Figure 8D). While the difference did not reach statistical significance, similarly to the spleen, we saw a reduced frequency of lung IL-4 secreting T cells in infected pregnant mice at 14 DPI (Figure 8E). Further, we saw 7.4-fold lower frequency of IL-10 secreting T cells in the lungs of pregnant mice compared to non-pregnant animals (*p* = 0.025) (Figure 8F). These data suggest pregnancy reduces the regulatory landscape of the lungs, potentially explaining the exacerbated pathology observed here. Additionally, enhanced systemic IL-4 levels in infected pregnant mice 10 DPI suggests potential for ongoing type-2 adaptive immune stimulation, which has been reported to exacerbate influenza infection (67).

### Delivery Potentiates Continued Antibody Class-Switch With Reduced Antibody Quality Which Resolves by 8 Weeks Post Delivery

**Figure 9:**
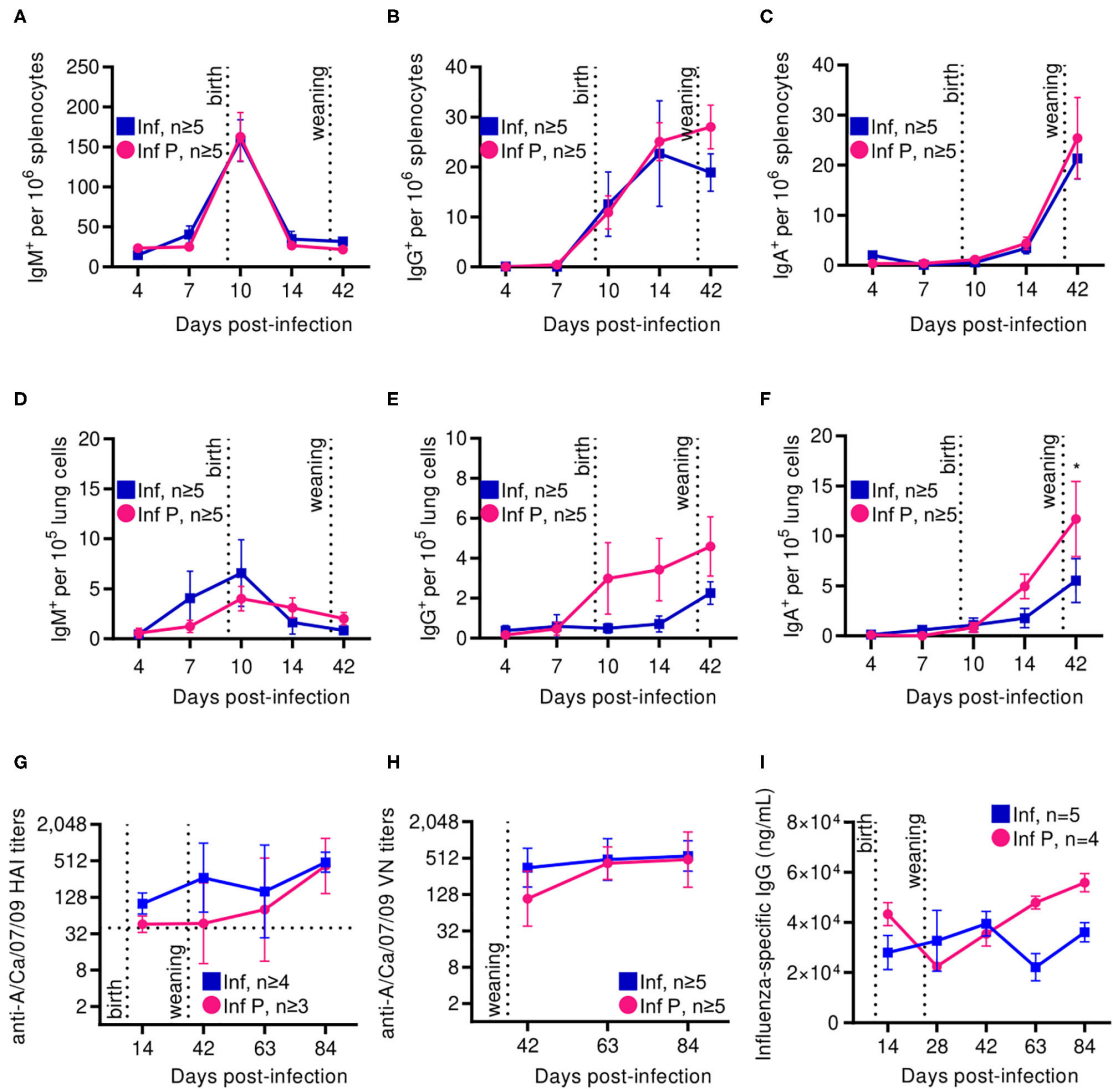
Following delivery, pregnant mice show increased ASCs in spleen and lungs, although these antibodies have lower neutralization and avidity. ASCs were quantified by ELISpot for **(A)** IgA, **(B)** IgM, and **(C)** IgG secretion among splenocytes at days 4, 7, 10, 14, and 42 DPI. ASCs were also quantified for **(D)** IgA, **(E)** IgM, and **(F)** IgG within lungs at the same time points. **(G)** A/California/07/2009-specific HAI titers were quantified at 14, 42, 63, and 84 DPI. **(H)** Virus neutralization titers from infected pregnant and non-pregnant animals were assessed against A/California/07/2009 by TCID50. **(I)** Total IgG titers were determined by ELISA against recombinant HA. All data was analyzed via Two-way ANOVA using Sidak correction for multiple comparisons. Error bars represent Standard Error of Mean (SEM) and mean for **(A–F)**, and geometric mean with gSD for **(G,H)**. *p < 0.05.

Progesterone is an established negative regulator of B cell activation (25). Additionally, pregnancy has been reported to both attenuate responses to influenza vaccination and to have no such effect (25). Therefore, we examined systemic and local antibody secreting cells (ASCs) in the spleen and lungs and further interrogated humoral immune responses by evaluating long-term antibody kinetics and quality. There were no differences between infected pregnant and non-pregnant cohorts in the kinetics and magnitude of IgM, IgG and IgA secreting cells from the spleen up to 42 DPI (Figures 9A–C). In the lungs however, non-pregnant animals tended to have a greater number of IgM secreting cells soon after infection (≤ 10D DPI) (Figure 9D), while pregnant animals had greater numbers of lung IgG-secreting cells as early as 10 DPI (Figure 9E). IgA-secreting cells reached a peak at 42 DPI, with 2 -fold higher counts in the pregnant infected animals (*p* = 0.034) (Figure 9F). No differences were observed in bone marrow IgG secreting cells, or in total serum IgG (data not shown). This data suggests that labor and nursing in pregnant animals may potentiate antibody class-switch for extended periods.

As hemagglutinin (HA) is indispensable to influenza binding to cellular sialic acids and therefore viral entry, we determined the level of hemagglutination inhibition by influenza virus-specific antibodies developed in pregnant and non-pregnant animals. By 14 DPI, both pregnant and non-pregnant animals had A/California/07/2009-specific HAI titers of at least 40, which correlate with protection. These titers continued rising in both groups through 42 DPI. Pregnant mice however showed delayed increases compared to non-pregnant controls, although endpoint titers at 84 DPI were comparable at 592 and 512, respectively (Figure 9G). Virus neutralization assays showed similar trends: both pregnant and non-pregnant animals had similar endpoint antibody neutralization titers 84 DPI, although at 42 DPI these titers were 480 and 608, respectively, replicating our previous observations of delayed antibody responses in pregnancy (Figure 9H). These alterations in antibody functionality are not a consequence of differential antibody titers, as IgG titers did not differ between pregnant and non-pregnant animals during our time course (Figure 9I).

**Figure 10:**
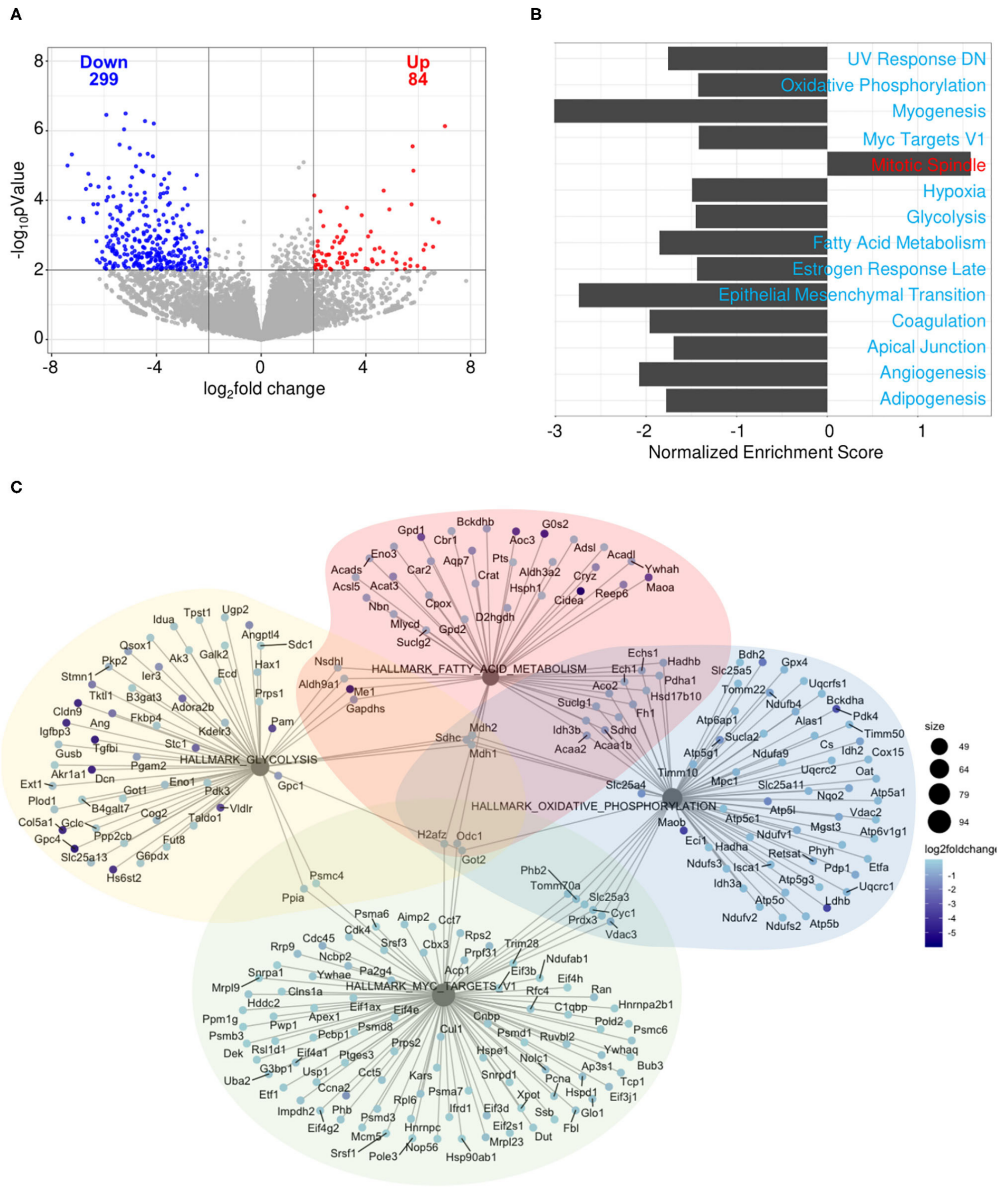
Splenic plasmablasts derived from pregnant mice downregulate metabolic gene expression. Splenic plasmablasts from pregnant and non-pregnant infected control mice at 10 DPI were sequenced and **(A)** upregulated and downregulated genes identified based on an adjusted *p*-value threshold of 0.01 and log2 fold change threshold of 2. Each dot represents one gene. The vertical line is drawn at 0 fold change, while the horizontal line is at the log10 (*p*-value = 0.01). Gene-set enrichment analysis (GSEA) **(B)** was performed using the hallmark gene sets from the MSigDB collection. Genes were ranked based on log2 fold change/standard error prior to running GSEA in pre-ranked mode, with padj < 0.05. Network plot **(C)** of gene sets involved in cellular metabolism. The size of each node represents the size of the gene set. Each gene is colored based on the log2 fold change. *p < 0.05.

To determine how gene expression in B cells is affected by pregnancy during an influenza infection, splenic plasmablasts from pregnant and non-pregnant infected mice collected at 10 DPI were sequenced. Pregnant mice displayed 84 upregulated genes and 299 downregulated genes based on an adjusted *p*-value threshold of 0.01 and log_2_ fold change threshold of 2 (Figure 10A). We performed gene-set enrichment analysis (GSEA) using the hallmark gene sets from the MSigDB collection to determine which pathways were associated with influenza virus infection during pregnancy. Genes were ranked based on log2 fold change/standard error prior to running GSEA in pre-ranked mode. Of the 50 gene sets within the hallmark gene set, 14 were significant (p_adj_ < 0.05). Only one gene set was positively enriched, the mitotic spindle gene set, the members of which are involved in mitotic spindle assembly (Figure 10B). Gene sets that were negatively enriched include myogenesis, UV response DN, hypoxia, estrogen response late, coagulation, apical junction, epithelial mesenchymal transition, angiogenesis and adipogenesis. Additionally, we found four gene sets involved in cellular metabolism and proliferation were negatively regulated in pregnant mice (Figure 10C). These data suggest that splenic plasmablasts derived from pregnant mice exhibit a metabolic deficiency during influenza infection which is not seen in non-pregnant infected animals and may be responsible for acutely delayed antibody quality.

## Discussion

The enhanced morbidity and mortality seen in pregnant women infected with the swine origin 2009 pandemic influenza virus triggered investigations into the underlying mechanisms of increased risk. Reports indicate increased mortality in pregnant mice and their offspring exposed to A/California/07/2009, enhanced lung immunopathology, and dysregulated progesterone and estrogen expression (13, 36, 68). In this study we confirm that infection of pregnant mice at mid-gestation with the pandemic H1N1 virus enhances maternal morbidity, and increases pre-term labor, incidence of SGA offspring and stillbirth, recapitulating clinical observations in pregnant populations. Our findings validate our previous observations of seasonal influenza virus-induced adverse outcomes in BALB/c mice following H1N1 A/Brisbane/59/2007 infection. We also demonstrate that pregnancy complications caused by the pandemic strain can be achieved at much lower infectious doses than the seasonal virus (0.5 × LD_50_ vs. 2 × LD_50_). Importantly, we expand upon previous work to evaluate the quality of adaptive immune responses. How the condition of pregnancy impacts long-term maternal anti-influenza immunity is contentious, and published research on adaptive immune responses in animal models of influenza during pregnancy is limited. Imbalanced antibodies in pregnant women hospitalized with 2009 H1N1 (43) and reports that influenza-infection-induced antibody titers are lower in pregnant mice (36) suggest that there may be differences to humoral immune responses to influenza during pregnancy. We confirm acutely delayed antibody quality which resolves 8 weeks after delivery in pregnant mice infected with pandemic influenza, and explore for transcriptional mechanisms implicated in these perturbations.

We observed worsened pulmonary pathology in pregnant infected mice compared to non-pregnant infected controls which coincided with higher viral loads, suggesting uncontrolled viral replication contributing to disease as previously described (36). We also detected increased numbers of occulted blood vessels and mucus-laden bronchioles, and exaggerated hyperplasia of goblet cells resulting in lung pathology more severe than non-pregnant counterparts, recapitulating described mechanisms of pregnancy-specific lung pathogenesis (36). In parallel, excess inflammation through upregulated IL-6, RANTES and MCP-1 and resulting increased recruitment of neutrophil and macrophages (12, 37) was implicated in enhanced lung pathology. These cytokines were highly expressed in humans who died as a result of 2009 H1N1 influenza A virus (69). Resulting recruitment of tissue damaging neutrophils and monocytes secreting remodeling enzymes such as MMPs, which have been highly correlated with acute lung injury (70), were also observed in our study. Indeed, it has been suggested that excessive production of inflammatory cytokines and chemokines may contribute to enhanced disease severity caused by influenza virus (15). Importantly, we did not observe recruitment of dendritic cells or T cells to the lungs of mice infected during pregnancy (37, 42). Lung progesterone in pregnant infected mice was reduced compared to uninfected pregnant counterparts; this loss of relaxation of maternal airway smooth muscles and bronchial tissue dilation (55) coincided with elevated PGF2α, which is known to cause bronchoconstriction (57). Elevated lung PGF2α was maintained even after elimination of virus, suggesting chronic lung abnormalities in infected animals independent of pregnancy status. Lastly, we observed altered ratios of glycosylated:unglycosylated COX-2, pointing to an infection-specific inhibition of post-translational modification in both pregnant and non-pregnant infected groups.

We next evaluated influenza-induced alterations to immune mediators as a connection between the site of influenza infection and local, placental perturbations. The cytokine profile correlated with increased severity of infection as previously reported (36, 43). Pregnant infected mice had substantial upregulation of inflammatory cytokines including TNF-α and IL-6 (12). IL-1β secretion, which contributes to inflammasome activation and has been implicated in influenza pathology (71), was increased in infected pregnant mice. We also observed increased levels of IL-10 in lungs of infected animals; influenza infection has been demonstrated to enhance the production of IL-10, thereby suppressing the overall immune response and promoting susceptibility to secondary infections (72).

Previous work by our group demonstrated that dysregulation of progesterone, COX-2, and prostaglandins in combination with upregulation of MMP9 at the placenta were key mechanisms by which a seasonal H1N1 virus, A/Brisbane/59/2007, induces adverse pregnancy outcomes (12). Those findings correlated with pathological remodeling of placental architecture, which culminated in a pro-abortive mechanism (12). Here, we further established that adverse outcomes on maternal and fetal health are brought about by indirect causes such as dysregulated hormone signaling and loss of placental architecture following infection with pandemic H1N1 A/California/07/2009. While progesterone is known to increase continually during the course of pregnancy (73), progesterone levels were reduced following influenza virus infection compared to uninfected controls in both lungs and placenta. Similarly to progesterone, placental PIBF increases throughout gestation (73) whereas reduction of functional isoforms due to infection may contribute to adverse pregnancy outcomes by disrupting the tight control of T cell and NK cell activity at the maternal-fetal interphase (26). Indeed, NK cell activation in the maternal decidua is a known complication of influenza infection in pregnancy (2). Influenza virus infection led to increased levels of placental PGF2α, a known progesterone down-regulator (74); our data confirmed the correlation of PGF2α with a significant reduction in progesterone among infected pregnant animals when compared to uninfected controls, likely resulting in reduced PIBF production (64). This infection-induced, PGF2α-mediated drop in progesterone and PIBF has been directly linked to miscarriage and gestation complications (75). Interestingly, following infection with A/California/07/2009, we observed differential regulation of PIBF isoforms in the placenta as compared with infections with A/Brisbane/59/2007 suggesting a strain specific effect.

Placentae from infected mothers demonstrated significant loss of architecture, particularly between the maternal and fetal layers, suggesting poor placental health. Both the placenta and the surrounding fetal membrane were subjected to enhanced MMP-mediated degradation quickly after influenza infection, as previously reported (12). Unlike previous reports of cultured human fetal membrane cells infected with influenza virus, we did not observe cytokine-mediated inflammation of the placenta (76); however, we did observe increases in monocyte-differentiation-inducing factors and chemokines responsible for the recruitment of T cells and macrophages such as RANTES and MCP-1α (77, 78). Indeed, 2 days following infection, pregnant infected mice had increased T cell and macrophage and numbers in their uterus. Further, infection increased neutrophils at the placenta of both live and stillborn pups, and placentae from stillborn pups had increased numbers of monocytes and T cells.

Dysregulated cytokine and chemokine production, excessive immune cell influx, and damage to the lungs are hypothesized to underlie influenza pathology (15, 16), but there is a paucity of data on immune responses to influenza virus infection during and following pregnancy. To understand the long-term consequences of influenza infection during pregnancy, we investigated B cell responses during pregnancy, while mothers were nursing, and after weaning. While all infected pregnant and non-pregnant animals reached protective HAI titers of ≥40, pregnant mice showed delayed production of functional antibodies compared to non-pregnant controls. Characterization of virus neutralizing capacity and binding strength of influenza-specific antibodies showed that animals infected during pregnancy had lower neutralizing antibody titers than non-pregnant counterparts until 63 DPI. As asymmetrically glycosylated IgG antibodies are increased in maternal serum and placental tissue during normal human pregnancy (79), this pregnancy-specific change in antibody quality could account for delayed functional antibodies, as it resolves following delivery.

RNA sequencing data of splenic plasmablasts revealed downregulated genes that were related to metabolic functions, such as glycolysis, oxidative phosphorylation, and fatty acid metabolism. These three pathways play direct roles in B cell survival, differentiation, and in antibody magnitude and quality as they undergo post-translational modifications, such as oxidation, phosphorylation, and glycosylation (80). Our observations of antibody hemagglutinin inhibition and virus neutralization which resolved within 8 weeks of delivery demonstrate that influenza infection impacts antibody maturation mechanisms without alterations to B cell frequency or antibody secretion. This hypothesis was further supported by our RNAseq data, which demonstrated that influenza virus infection directly down-regulates B cell metabolism and post-translational modification systems among pregnant animals, providing a potential mechanism for resolving antibody deficiencies. Previous studies have identified additional humoral immunity perturbations in pregnant women infected with influenza virus including lower circulating levels of IgG2 antibody (43), which at high levels is hypothesized to protect against secondary bacterial infections following influenza infection (42). Indeed, reduced levels of IgG2 are associated with severe health outcome and dysregulated cytokine production (41).

These humoral immune perturbations suggest potential complications for influenza vaccines administered during pregnancy; however, additional studies are necessary in order to identify mechanisms responsible for the observed resolving antibody deficiencies. While influenza vaccines have been reported to be safe and effective at preventing influenza illness in pregnant women and their newborn children for several months after birth (2), clinical data is inconclusive regarding the efficiency of immune responses to the vaccine when compared to those induced in non-pregnant women. Richardson et al. reported that, after H1N1 vaccination, pregnant women had increased regulatory T cells and decreased antibody responses (81). Additionally, Schlaudecker et al. reported that HAI titers were reduced in pregnant women after immunization for A/California/07/2009 and H3N2 A/Perth/16/2009 (47). These reports conflicted with others reporting no loss of protection following influenza vaccination during pregnancy (2). Future studies should explore mechanisms for these discrepancies and ensure use of non-morbid models for the study of long-term humoral immunity alterations.

Collectively, our findings support two interlocking hypotheses. First, the immunological alterations that occur during pregnancy to maintain fetal-maternal homeostasis allow for exacerbated influenza pathogenesis, ultimately contributing to shortened gestation length and impaired fetal outcomes. Previous work by our group using a seasonal A/Brisbane/59/2007 strain of influenza supported this conclusion and also suggested that infection during pregnancy may indirectly affect B cells and antibodies (12). This study used a pandemic A/California/07/2009 strain to induce a more pathogenic phenotype in order to investigate this second hypothesis further. Our data suggests that antibody anomalies observed during influenza infection are a result of the immunomodulatory impact of pregnancy impairing B cell responses, and not a result of infection *per se*. We observed lower antibody neutralizing titers but no changes in ASC frequencies during pregnancy, and transcriptomic analysis of these ASCs revealed that plasmablast metabolism and post-translational modifications were down-regulated among pregnant mice only. In conclusion, our findings propose a link between adverse pregnancy outcomes and severe lung pathology observed during influenza infection with plasmablast metabolic dysregulation leading to poor antibody quality. Enhanced understanding of pregnancy-specific factors influencing interaction of the virus with host humoral immunity is important for the development of more effective prevention and treatment options in the future.

## Data Availability Statement

Original datasets are available in a publicly accessible repository: The original contributions presented in the study are publicly available. This data can be found here: GSE155388.

## Ethics Statement

The animal study was reviewed and approved by Animal studies were conducted according to Emory University Institutional Animal Care and Use Committee (IACUC) guidelines outlined in an approved protocol (PROTO201800113) in compliance with the United States Federal Animal Welfare Act (PL 89–544) and subsequent amendments.

## Author Contributions

EL and IS conceived of and planned experiments. EL, KB, EE, LM, DW, and OA carried out experiments. EL, DS, LM, JB, and KB contributed to the interpretation of the results. DS wrote the manuscript. All authors provided critical feedback and helped shape the research, analysis, and manuscript.

## Conflict of Interest

The authors declare that the research was conducted in the absence of any commercial or financial relationships that could be construed as a potential conflict of interest.
