# Supplementary material for: Pregnancy Downregulates Plasmablast Metabolic Gene Expression Following Influenza Without Altering Long-Term Antibody Function
Source: Front Immunol. 2020 Aug 14;11:1785. doi: 10.3389/fimmu.2020.01785 (PMC7457062; doi:10.3389/fimmu.2020.01785)
Supplement: Supplementary file 1 [file Data_Sheet_1.PDF]

| | Nonpregnant | | | | Pregnant | | | | P/NP | |
|---|---|---|---|---|---|---|---|---|---|---|
| | Uninfected | Infected | Fold Change* | q | Uninfected | Infected | Fold Change | q | Fold Change* | q |
| **IL-2** | 12470.4 ± 683.5 | 12897.2 ± 1951.1 | 1.0 | 0.91 | 14009.5 ± 247.4 | 10182.4 ± 667.6 | -1.4 | 0.06 | -1.3 | 0.40 |
| **IL-9** | 18751.9 ± 2519.9 | 18343.7 ± 2454.7 | -1.0 | 0.92 | 22380.9 ± 1281.2 | 13913.1 ± 1744.8 | -1.6 | 0.09 | -1.3 | 0.34 |
| **IL-3** | 5238.4 ± 370.2 | 6176.5 ± 1146 | 1.2 | 0.64 | 5280.9 ± 157.8 | 5200.9 ± 383.9 | -1.0 | 0.91 | -1.2 | 0.62 |
| **IL-5** | 7460.9 ± 526 | 7176.5 ± 1182.6 | -1.0 | 0.91 | 7004.7 ± 169 | 4457.8 ± 800.7 | -1.6 | 0.16 | -1.6 | 0.22 |
| **G-CSF** | 3373.6 ± 232.9 | 57383 ± 15342.7 | 17.0 | 0.09 | 4376.1 ± 85.8 | 60293 ± 9442.8 | 13.8 | 0.05 | 1.1 | 0.91 |
| **GM-CSF** | 8382.3 ± 426.8 | 13414 ± 2680 | 1.6 | 0.29 | 8980.9 ± 338.9 | 10052.9 ± 1120.8 | 1.1 | 0.64 | -1.3 | 0.45 |
| **IL-1α** | 14673.4 ± 1123.7 | 24672.4 ± 5028.8 | 1.7 | 0.27 | 16552.3 ± 554.5 | 27231.6 ± 3963.8 | 1.6 | 0.22 | 1.1 | 0.80 |
| **IL-1β** | 9604.5 ± 771.4 | 11071.2 ± 2111.8 | 1.2 | 0.69 | 11676.1 ± 208.8 | 6981.6 ± 728 | -1.7 | 0.04 | -1.6 | 0.24 |
| **IL-6** | 8677.5 ± 517.4 | 167683.6 ± 54854.9 | 19.3 | 0.13 | 9957.1 ± 250.8 | 70530.2 ± 14025.4 | 7.1 | 0.09 | -2.4 | 0.27 |
| **IL-12(p70)** | 8686.2 ± 645.3 | 11663.8 ± 2622.7 | 1.3 | 0.53 | 9061.9 ± 261.9 | 8629.9 ± 730 | -1.1 | 0.78 | -1.4 | 0.48 |
| **IL-17** | 10786.1 ± 860 | 12793.7 ± 2417.4 | 1.2 | 0.64 | 14047.6 ± 272.2 | 11036.2 ± 732 | -1.3 | 0.10 | -1.2 | 0.64 |
| **IFN-γ** | 9236.7 ± 667 | 13222.2 ± 2419.6 | 1.4 | 0.37 | 9461.9 ± 246.9 | 7926.5 ± 418.1 | -1.2 | 0.13 | -1.7 | 0.18 |
| **TNF-α** | 5048.1 ± 539.1 | 5286.7 ± 884.5 | 1.0 | 0.91 | 5490.4 ± 217.8 | 4060.1 ± 424.2 | -1.4 | 0.15 | -1.3 | 0.41 |
| **IL-4** | 2244.5 ± 135.4 | 3838.5 ± 842.6 | 1.7 | 0.29 | 4795.2 ± 12.5 | 3303.3 ± 379.2 | -1.5 | 0.10 | -1.2 | 0.69 |
| **IL-10** | 5844.9 ± 504.9 | 7533.2 ± 1739.8 | 1.3 | 0.61 | 6219 ± 295.5 | 5234.6 ± 411.8 | -1.2 | 0.29 | -1.4 | 0.40 |
| **IL-13** | 8947.9 ± 744.5 | 8677.5 ± 1471.1 | -1.0 | 0.91 | 9642.8 ± 678.5 | 5823 ± 719.9 | -1.7 | 0.08 | -1.5 | 0.26 |
| **Eotaxin** | 10940.3 ± 1513.5 | 13113.2 ± 2379.7 | 1.2 | 0.64 | 12771.4 ± 882.6 | 8877.7 ± 1386.5 | -1.4 | 0.22 | -1.5 | 0.31 |
| **KC** | 13457.6 ± 947 | 369929.7 ± 113209.5 | 27.5 | 0.10 | 35014.2 ± 1347.1 | 238626.2 ± 43202.2 | 6.8 | 0.08 | -1.6 | 0.49 |
| **MCP-1** | 3237 ± 163.8 | 70509.4 ± 18578.8 | 21.8 | 0.09 | 5695.2 ± 66.6 | 64325.7 ± 16607 | 11.3 | 0.13 | -1.1 | 0.89 |
| **MIP-1α** | 39455.8 ± 1582.9 | 265706.7 ± 65716.6 | 6.7 | 0.09 | 61414.2 ± 611.2 | 155627.9 ± 22905.1 | 2.5 | 0.10 | -1.7 | 0.30 |
| **MIP-1β** | 15531.2 ± 1201.2 | 58773.7 ± 13522.9 | 3.8 | 0.10 | 18961.9 ± 361.9 | 33651 ± 5236.5 | 1.8 | 0.20 | -1.7 | 0.26 |
| **RANTES** | 454098.8 ± 47642.6 | 826791.7 ± 165093.3 | 1.8 | 0.22 | 373609.5 ± 11592.5 | 599480.8 ± 46690.1 | 1.6 | 0.08 | -1.4 | 0.40 |
| **IL-12(p40)** | 11810.6 ± 935.6 | 195755.4 ± 73700.1 | 16.6 | 0.18 | 13804.7 ± 189.9 | 97014.3 ± 17100.3 | 7.0 | 0.08 | -2.0 | 0.40 |

**Supplementary Table 1: Lung chemokine and cytokine levels 4 days post-infection.**

Protein concentrations from infected and uninfected lung lysates collected at 4 DPI. Lysates were quantified for growth factors, inflammatory and anti-inflammatory cytokine, and chemokine concentrations. *Fold change was transformed as follows: if fold change >1, no transformation; if fold change <1, - ($10^{|\log10 \text{fold change}|}$). The shaded fold-differences are significant (q<0.05). Cytokine quantitation was analyzed via Two-way ANOVA and post-hoc multiple T-tests without assuming consistent SD with correction for multiple comparisons by controlling the false discovery rate per the two-stage set up method of Benjamini Krieger and Yekutieli (Q=5%).
