# Supplementary material for: Pregnancy Downregulates Plasmablast Metabolic Gene Expression Following Influenza Without Altering Long-Term Antibody Function
Source: Front Immunol. 2020 Aug 14;11:1785. doi: 10.3389/fimmu.2020.01785 (PMC7457062; doi:10.3389/fimmu.2020.01785)
Supplement: Supplementary file 2 [file Data_Sheet_2.PDF]

	Nonpregnant				Pregnant				P/NP	
	Uninfected	Infected	Fold Change*	q	Uninfected	Infected	Fold Change	q	Fold Change*	q
**IL-2**	12470.4 ± 683.5	12142 ± 1996.4	-1.0	0.91	12966.6 ± 1482.5	11972.5 ± 1860.4	-1.1	0.81	-1.0	0.94
**IL-9**	18751.9 ± 2519.9	13815.9 ± 2410.6	-1.4	0.37	17411.1 ± 1719.4	16798 ± 4262.2	-1.0	0.92	1.2	0.68
**IL-3**	5238.4 ± 370.2	6707.8 ± 948.2	1.3	0.40	4012.5 ± 481.3	8369.9 ± 814.1	2.1	0.08	1.2	0.40
**IL-5**	7460.9 ± 526	13449.6 ± 7067.6	1.8	0.64	7369.4 ± 1461.4	9221.1 ± 1087.8	1.3	0.52	-1.5	0.69
**G-CSF**	3373.6 ± 232.9	57396.2 ± 7111.8	17.0	<0.01	3043 ± 320.7	120913.2 ± 23664.3	39.7	0.08	2.1	0.12
**GM-CSF**	8382.3 ± 426.8	9707.5 ± 1216.2	1.2	0.56	7348.6 ± 942.1	11316.1 ± 1634.2	1.5	0.28	1.2	0.63
**IL-1α**	14673.4 ± 1123.7	19802.1 ± 2402.8	1.3	0.26	13537.5 ± 1251.6	26893.3 ± 1476.2	2.0	0.02	1.4	0.12
**IL-1β**	9604.5 ± 771.4	10842.1 ± 1641.7	1.1	0.68	8408.3 ± 74	10792.8 ± 1464	1.3	0.44	-1.0	0.96
**IL-6**	8677.5 ± 517.4	121962.4 ± 46179.3	14.1	0.18	8636.1 ± 1084.1	173568.1 ± 36277.7	20.1	0.09	1.4	0.58
**IL-12(p70)**	8686.2 ± 645.3	11781.4 ± 1325.4	1.4	0.22	7830.5 ± 382.5	13372.7 ± 1657.3	1.7	0.15	1.1	0.64
**IL-17**	10786.1 ± 860	11439.6 ± 1359.3	1.1	0.81	10373.6 ± 1032.6	13413.8 ± 2298.9	1.3	0.55	1.2	0.64
**IFN-γ**	9236.7 ± 667	68553.7 ± 25105.6	7.4	0.20	8883.3 ± 635.1	75673.9 ± 23469.7	8.5	0.20	1.1	0.91
**TNF-α**	5048.1 ± 539.1	4625.7 ± 551.7	-1.1	0.71	4750 ± 573.5	5421.2 ± 715.8	1.1	0.68	1.2	0.58
**IL-4**	2244.5 ± 135.4	5083 ± 881.9	2.3	0.10	2416.6 ± 171.7	6323.2 ± 761.8	2.6	0.08	1.2	0.49
**IL-10**	5844.9 ± 504.9	25115.4 ± 6470.8	4.3	0.12	5366.6 ± 263.6	24592.6 ± 2935.5	4.6	0.04	-1.0	0.94
**IL-13**	8947.9 ± 744.5	8172.7 ± 1074	-1.1	0.70	7905.5 ± 357.1	7631.1 ± 1268.6	-1.0	0.91	-1.1	0.84
**Eotaxin**	10940.3 ± 1513.5	9776.9 ± 1147.3	-1.1	0.68	8969.4 ± 810.2	12243 ± 2171.7	1.4	0.49	1.3	0.52
**KC**	13457.6 ± 947	154905.7 ± 43806.4	11.5	0.10	17412.5 ± 3202.1	156443.1 ± 42441.8	9.0	0.15	1.0	0.96
**MCP-1**	3237 ± 163.8	101245.7 ± 28329.5	31.3	0.09	3004.1 ± 223.1	243195.5 ± 33617.3	81.0	0.03	2.4	0.08
**MIP-1α**	39455.8 ± 1582.9	529329.4 ± 74771.3	13.4	0.02	28183.3 ± 4810.9	491407.7 ± 27024.1	17.4	<0.01	-1.1	0.75
**MIP-1β**	15531.2 ± 1201.2	180193.2 ± 37619.4	11.6	0.06	13259.7 ± 1010.1	150194.6 ± 10524.7	11.3	<0.01	-1.2	0.64
**RANTES**	454098.8 ± 47642.6	645769.2 ± 72087.5	1.4	0.20	186470.8 ± 74356.3	665643 ± 100838.2	3.6	0.09	1.0	0.91
**IL-12(p40)**	11810.6 ± 935.6	81709 ± 4828.9	6.9	<0.01	10050 ± 1576	69853.3 ± 8383.4	7.0	0.03	-1.2	0.42

Supplementary Table 2: Lung chemokine and cytokine levels 7 days post-infection.

Protein concentrations from infected and uninfected lung lysates collected at 7 DPI. Lysates were quantified for growth factors, inflammatory and anti-inflammatory cytokine, and chemokine concentrations. *Fold change was transformed as follows: if fold change >1, no transformation; if fold change <1, - ($10^{|\text{log10fold change}|}$). The shaded fold-differences are significant (q<0.05). Cytokine quantitation was analyzed via Two-way ANOVA and post-hoc multiple T-tests without assuming consistent SD with correction for multiple comparisons by controlling the false discovery rate per the two-stage set up method of Benjamini Krieger and Yekutieli (Q=5%).
