# Supplementary material for: Pregnancy Downregulates Plasmablast Metabolic Gene Expression Following Influenza Without Altering Long-Term Antibody Function
Source: Front Immunol. 2020 Aug 14;11:1785. doi: 10.3389/fimmu.2020.01785 (PMC7457062; doi:10.3389/fimmu.2020.01785)
Supplement: Supplementary file 3 [file Data_Sheet_3.PDF]

	Nonpregnant				Pregnant				P/NP	
	Uninfected	Infected	Fold Change*	q	Uninfected	Infected	Fold Change	q	Fold Change*	q
**IL-2**	1123 ± 128.6	945 ± 131	-1.2	0.81	806 ± 41.1	1127.5 ± 278.7	1.4	0.81	1.2	0.84
**IL-9**	2728.5 ± 1180.3	1647 ± 263.2	-1.7	0.81	997.3 ± 88.7	1037 ± 231.8	1.0	>0.99	-1.6	0.79
**IL-3**	1634.5 ± 685.9	1075 ± 26.4	-1.5	0.81	1172 ± 135.2	1248 ± 224.7	1.1	0.99	1.2	0.81
**IL-5**	1839 ± 976.2	1197.5 ± 138.3	-1.5	0.83	1393.3 ± 271.1	1113.5 ± 166.5	-1.3	0.81	-1.1	0.95
**G-CSF**	2943 ± 587.4	5336 ± 841	1.8	0.79	2393.3 ± 237	5404.5 ± 884.8	2.3	0.76	1.0	>0.99
**GM-CSF**	3165 ± 1576.9	1899.5 ± 150.3	-1.7	0.81	2026 ± 217.9	1667.5 ± 201.1	-1.2	0.81	-1.1	0.81
**IL-1α**	1637.5 ± 138.9	1309 ± 127.8	-1.3	0.79	830.6 ± 101.8	1296 ± 84.8	1.6	0.76	-1.0	>0.99
**IL-1β**	1842 ± 1254.5	1018.5 ± 100.1	-1.8	0.83	1120.6 ± 283.9	731.5 ± 118.3	-1.5	0.81	-1.4	0.79
**IL-6**	2820 ± 1553.5	2124.5 ± 104.8	-1.3	0.91	2100 ± 498	2024.5 ± 373.7	-1.0	>0.99	-1.0	0.99
**IL-12(p70)**	4344 ± 1603.5	2380 ± 297.4	-1.8	0.81	3650 ± 67.3	3408 ± 629.8	-1.1	0.98	1.4	0.80
**IL-17**	9618 ± 1077.7	7653 ± 1028.5	-1.3	0.81	15038.6 ± 1848.1	16946.5 ± 4083.9	1.1	0.95	2.2	0.79
**IFN-γ**	5319.5 ± 2685.5	3042 ± 308.8	-1.7	0.81	4363.3 ± 616.7	2961.5 ± 581.5	-1.5	0.79	-1.0	>0.99
**TNF-α**	4193 ± 2346.3	2439 ± 305.1	-1.7	0.81	3373.3 ± 527.7	2380 ± 547.7	-1.4	0.81	-1.0	>0.99
**IL-4**	1451.5 ± 771.6	786.5 ± 49.7	-1.8	0.81	1045.3 ± 225.8	751.5 ± 150.3	-1.4	0.81	-1.0	>0.99
**IL-10**	2289 ± 1432.7	1250.5 ± 110.7	-1.8	0.81	1744 ± 278.4	1378.5 ± 345.4	-1.3	0.81	1.1	0.96
**IL-13**	1549.5 ± 878.3	1003.5 ± 32.6	-1.5	0.84	1143.3 ± 206.5	966.5 ± 150.7	-1.2	0.81	-1.0	0.99
**Eotaxin**	2438 ± 1240.8	1515 ± 152.2	-1.6	0.81	1726.6 ± 359.4	1297.5 ± 191.2	-1.3	0.81	-1.2	0.81
**KC**	2772 ± 1391.6	2750.5 ± 270.9	-1.0	>0.99	2604.6 ± 579.6	3002.5 ± 202.5	1.2	0.81	1.1	0.81
**MCP-1**	2295 ± 1475.1	1244.5 ± 128.5	-1.8	0.81	1530 ± 354.1	983 ± 177.3	-1.6	0.80	-1.3	0.81
**MIP-1α**	6383.5 ± 2128.8	3495 ± 184.4	-1.8	0.81	4549.3 ± 386.3	4044.5 ± 299.7	-1.1	0.81	1.2	0.79
**MIP-1β**	2580.5 ± 1487.2	1773.5 ± 148.7	-1.5	0.87	1812 ± 391.6	1830.5 ± 394.1	1.0	>0.99	1.0	>0.99
**RANTES**	5180 ± 1778.3	6816.5 ± 648.9	1.3	0.81	4004 ± 332.7	4795 ± 1002.1	1.2	0.83	-1.4	0.79
**IL-12(p40)**	7169 ± 2465.5	12235.5 ± 789.4	1.7	0.79	5478.6 ± 1195.4	10289.5 ± 1119.3	1.9	0.76	-1.2	0.81

Supplementary Table 3: Serum chemokine and cytokine levels 4 days post-infection.

Protein concentrations from infected and uninfected sera collected at 4 DPI. Sera was quantified for growth factors, inflammatory and anti-inflammatory cytokine, and chemokine concentrations. *Fold change was transformed as follows: if fold change >1, no transformation; if fold change <1, - ($10^{|\log10 \text{fold change}|}$). The shaded fold-differences are significant ($q<0.05$). Cytokine quantitation was analyzed via Two-way ANOVA and post-hoc multiple T-tests without assuming consistent SD with correction for multiple comparisons by controlling the false discovery rate per the two-stage set up method of Benjamini Krieger and Yekutieli (Q=5%).
