# Supplementary material for: Pregnancy Downregulates Plasmablast Metabolic Gene Expression Following Influenza Without Altering Long-Term Antibody Function
Source: Front Immunol. 2020 Aug 14;11:1785. doi: 10.3389/fimmu.2020.01785 (PMC7457062; doi:10.3389/fimmu.2020.01785)
Supplement: Supplementary file 4 [file Data_Sheet_4.PDF]

	Nonpregnant				Pregnant				P/NP	
	Uninfected	Infected	Fold Change*	q	Uninfected	Infected	Fold Change	q	Fold Change*	q
**IL-2**	1123 ± 128.6	994.4 ± 117.8	-1.1	>0.99	1384.6 ± 126.7	1239.2 ± 137.1	-1.1	0.81	1.2	0.81
**IL-9**	2728.5 ± 1180.3	3057.2 ± 329.7	1.1	0.90	1086.6 ± 1.3	4835.6 ± 1016.7	4.4	0.76	1.6	0.81
**IL-3**	1634.5 ± 685.9	2673.6 ± 647.6	1.6	0.80	1780 ± 261.5	2590 ± 509.2	1.5	0.81	-1.0	0.84
**IL-5**	1839 ± 976.2	2663.2 ± 720	1.4	0.81	1082.6 ± 188.1	2943.2 ± 544.8	2.7	0.79	1.1	>0.99
**G-CSF**	2943 ± 587.4	4764.8 ± 829.3	1.6	0.79	3893.3 ± 189.5	6003.6 ± 342	1.5	0.64	1.3	0.81
**GM-CSF**	3165 ± 1576.9	4628 ± 1050.9	1.5	0.81	2048 ± 238.1	4684 ± 1103.7	2.3	0.79	1.0	0.93
**IL-1α**	1637.5 ± 138.9	1624.8 ± 204.9	-1.0	>0.99	1720 ± 479.3	3732 ± 1824.3	2.2	0.81	2.3	0.81
**IL-1β**	1842 ± 1254.5	3108.8 ± 971.4	1.7	0.81	890 ± 152.4	3934.4 ± 1409	4.4	0.79	1.3	>0.99
**IL-6**	2820 ± 1553.5	4610.4 ± 1160.1	1.6	0.81	2084 ± 221.6	5176 ± 973.9	2.5	0.79	1.1	>0.99
**IL-12(p70)**	4344 ± 1603.5	6193.6 ± 1386	1.4	0.81	5216 ± 679.2	6090.4 ± 997.5	1.2	0.84	-1.0	0.87
**IL-17**	9354.6 ± 1279.9	10565.6 ± 1567.1	1.1	0.79	24370.6 ± 3709.8	12228 ± 1423.5	-2.0	0.76	1.2	>0.99
**IFN-γ**	5319.5 ± 2685.5	7782.4 ± 1669.2	1.5	0.81	4052 ± 557.7	7626.4 ± 1453.5	1.9	0.79	-1.0	0.88
**TNF-α**	4193 ± 2346.3	6638.8 ± 1638.6	1.6	0.81	3192.6 ± 473.1	7473.6 ± 1713.4	2.3	0.79	1.1	>0.99
**IL-4**	1451.5 ± 771.6	2064.8 ± 466.5	1.4	0.81	1145.3 ± 45.3	2170.8 ± 518.5	1.9	0.80	1.1	0.99
**IL-10**	2289 ± 1432.7	3395.2 ± 916.5	1.5	0.81	1793.3 ± 240.8	3239.6 ± 621	1.8	0.79	-1.0	0.82
**IL-13**	1549.5 ± 878.3	2236.4 ± 518.8	1.4	0.81	1144 ± 89	2446.8 ± 466.4	2.1	0.79	1.1	>0.99
**Eotaxin**	2438 ± 1240.8	4129.6 ± 1037.6	1.7	0.81	1550 ± 168.7	3969.2 ± 828.2	2.6	0.79	-1.0	0.87
**KC**	2772 ± 1391.6	3602 ± 541.8	1.3	0.81	1918.6 ± 140.1	3607.2 ± 448.3	1.9	0.76	1.0	0.88
**MCP-1**	2295 ± 1475.1	3805.6 ± 1080.8	1.7	0.81	1276.6 ± 147.3	3998.4 ± 982.4	3.1	0.79	1.1	0.99
**MIP-1α**	6383.5 ± 2128.8	8038.8 ± 1481.5	1.3	0.81	4685.3 ± 313	8392.8 ± 1395.9	1.8	0.79	1.0	0.99
**MIP-1β**	2580.5 ± 1487.2	4271.6 ± 1183.4	1.7	0.81	2286 ± 237.7	4546 ± 822.1	2.0	0.79	1.1	0.99
**RANTES**	6906.6 ± 602	8347.6 ± 1286.7	1.2	0.81	6256.6 ± 1068.8	6719.2 ± 1230.8	1.1	0.99	-1.2	0.81
**IL-12(p40)**	7169 ± 2465.5	8864.8 ± 778.6	1.2	0.81	6916.6 ± 964.1	7646.4 ± 969.8	1.1	0.88	-1.2	0.79

Supplementary Table 4: Serum chemokine and cytokine levels 7 days post-infection.

Protein concentrations from infected and uninfected sera collected at 7 DPI. Sera was quantified for growth factors, inflammatory and anti-inflammatory cytokine, and chemokine concentrations. *Fold change was transformed as follows: if fold change >1, no transformation; if fold change <1, - ($10^{|\log10 \text{fold change}|}$). The shaded fold-differences are significant (q<0.05). Cytokine quantitation was analyzed via Two-way ANOVA and post-hoc multiple T-tests without assuming consistent SD with correction for multiple comparisons by controlling the false discovery rate per the two-stage set up method of Benjamini Krieger and Yekutieli (Q=5%).
