# Supplementary material for: Pregnancy Downregulates Plasmablast Metabolic Gene Expression Following Influenza Without Altering Long-Term Antibody Function
Source: Front Immunol. 2020 Aug 14;11:1785. doi: 10.3389/fimmu.2020.01785 (PMC7457062; doi:10.3389/fimmu.2020.01785)
Supplement: Supplementary file 5 [file Data_Sheet_5.PDF]

	4 DPI				7 DPI			
	Uninfected	Infected	Fold Change*	q	Uninfected	Infected	Fold Change*	q
**IL-2**	7674 ± 575.1	3904.3 ± 296.1	-2.0	<0.01	3088.8 ± 101.8	4388.5 ± 325.7	1.4	0.03
**IL-9**	9118.8 ± 2022.4	2693.2 ± 165.7	-3.4	0.04	4411.2 ± 144.2	5599.5 ± 417.5	1.3	0.06
**IL-3**	2252.4 ± 37.2	2526.3 ± 194.7	1.1	0.25	2270.4 ± 77.1	2488 ± 145.3	1.1	0.26
**IL-5**	1550.4 ± 53.5	1184.1 ± 68.5	-1.3	0.03	1098 ± 22.6	1212 ± 47	1.1	0.11
**G-CSF**	15913.2 ± 2668.9	33178.6 ± 4464.2	2.1	0.03	29504.4 ± 8299.3	22117.2 ± 6095.8	-1.3	0.47
**GM-CSF**	4114.8 ± 57.1	4086.4 ± 180.8	-1.0	0.68	4088.4 ± 126.8	4377.8 ± 161.7	1.1	0.26
**IL-1α**	31260 ± 2555.2	15575.9 ± 2479.8	-2.0	0.03	9589.2 ± 1630.8	8586 ± 505	-1.1	0.51
**IL-1β**	2462.4 ± 124.1	1789 ± 150.9	-1.4	0.03	1882.8 ± 51.8	2493.1 ± 301.8	1.3	0.12
**IL-6**	3144 ± 80.6	2640 ± 117.1	-1.2	0.03	2834.4 ± 116.1	3212.7 ± 156.9	1.1	0.14
**IL-12(p70)**	3660 ± 66.5	3872.8 ± 352.5	1.1	0.48	3391.2 ± 138.4	3563 ± 143	1.1	0.42
**IL-17**	4314 ± 185.7	3962.5 ± 421.7	-1.1	0.44	3910.8 ± 191.6	3555 ± 167.5	-1.1	0.26
**IFN-γ**	3895.2 ± 127.8	3499 ± 285.1	-1.1	0.26	3471.6 ± 122.1	4103.2 ± 237.5	1.2	0.09
**TNF-α**	1808.4 ± 97.5	1362.5 ± 68.7	-1.3	0.03	1294.8 ± 54.8	1455.5 ± 53.7	1.1	0.12
**IL-4**	979.2 ± 29.7	1003.8 ± 90	1.0	0.65	1062 ± 66.4	927.1 ± 40.1	-1.1	0.20
**IL-10**	1893.6 ± 57.9	1609.2 ± 94.3	-1.2	0.06	1674 ± 31.6	1743.3 ± 48.9	1.0	0.30
**IL-13**	3189.6 ± 137.6	1572.2 ± 67	-2.0	<0.01	1658.4 ± 66.2	2001.1 ± 109.4	1.2	0.06
**Eotaxin**	3897.6 ± 269.7	2737.3 ± 158.3	-1.4	0.03	3117.6 ± 214.8	5225.1 ± 489.2	1.7	0.03
**KC**	45151.2 ± 4996.1	52324.6 ± 6605.7	1.2	0.42	17089.2 ± 1814.4	37420.2 ± 8658.9	2.2	0.09
**MCP-1**	2234.4 ± 267.9	3315.2 ± 295.4	1.5	0.06	2481.6 ± 364.6	4222.1 ± 760.6	1.7	0.12
**MIP-1α**	16624.8 ± 955.9	30334.8 ± 4884.6	1.8	0.06	25503.6 ± 3339	23202.9 ± 739	-1.1	0.48
**MIP-1β**	4576.8 ± 329.5	4181.6 ± 328.5	-1.1	0.42	4530 ± 548.5	5089.2 ± 519	1.1	0.46
**RANTES**	12465.6 ± 1901.6	6981 ± 688.8	-1.8	0.06	6019.2 ± 418.8	8367.7 ± 378.2	1.4	0.03
**IL-12(p40)**	7386 ± 211.5	7448.8 ± 220	1.0	0.66	5636.4 ± 509.7	5673.7 ± 573.5	1.0	0.73

Supplementary Table 5: Serum chemokine and cytokine levels 4 and 7 days post-infection.

Protein concentrations from infected and uninfected placenta collected at 4 and 7 DPI. Lysates were quantified for growth factors, inflammatory and anti-inflammatory cytokine, and chemokine concentrations. *Fold change was transformed as follows: if fold change >1, no transformation; if fold change <1, - ($10^{|\log10 \text{fold change}|}$). The shaded fold-differences are significant (q<0.05). Cytokine quantitation was analyzed via Two-way ANOVA and post-hoc multiple T-tests without assuming consistent SD with correction for multiple comparisons by controlling the false discovery rate per the two-stage set up method of Benjamini Krieger and Yekutieli (Q=5%).
