# Supplementary figures and images for: Pregnancy Downregulates Plasmablast Metabolic Gene Expression Following Influenza Without Altering Long-Term Antibody Function
Source: Front Immunol. 2020 Aug 14;11:1785. doi: 10.3389/fimmu.2020.01785 (PMC7457062; doi:10.3389/fimmu.2020.01785)

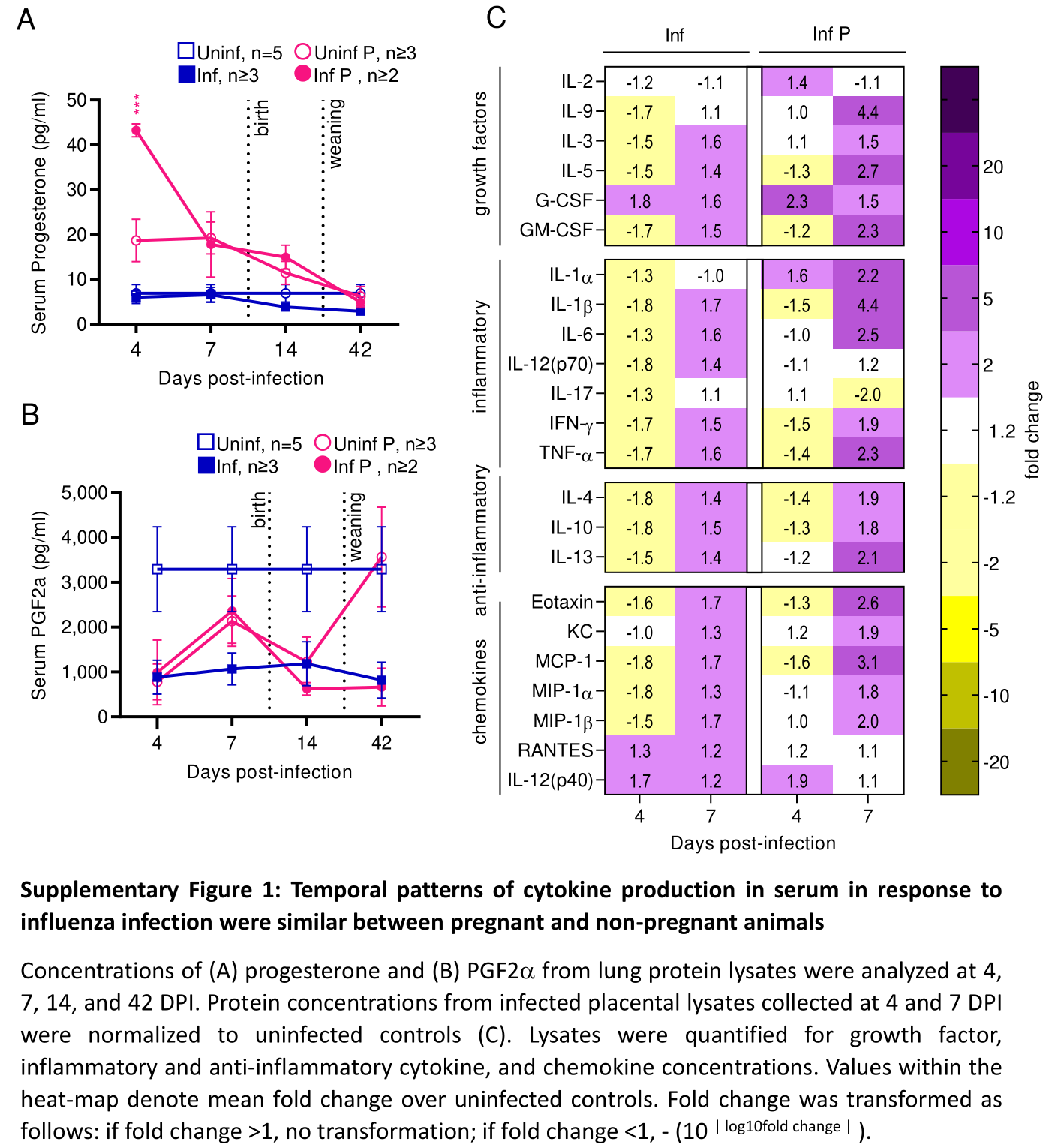

Supplement: Supplementary file 6 [file Image_1.TIFF]

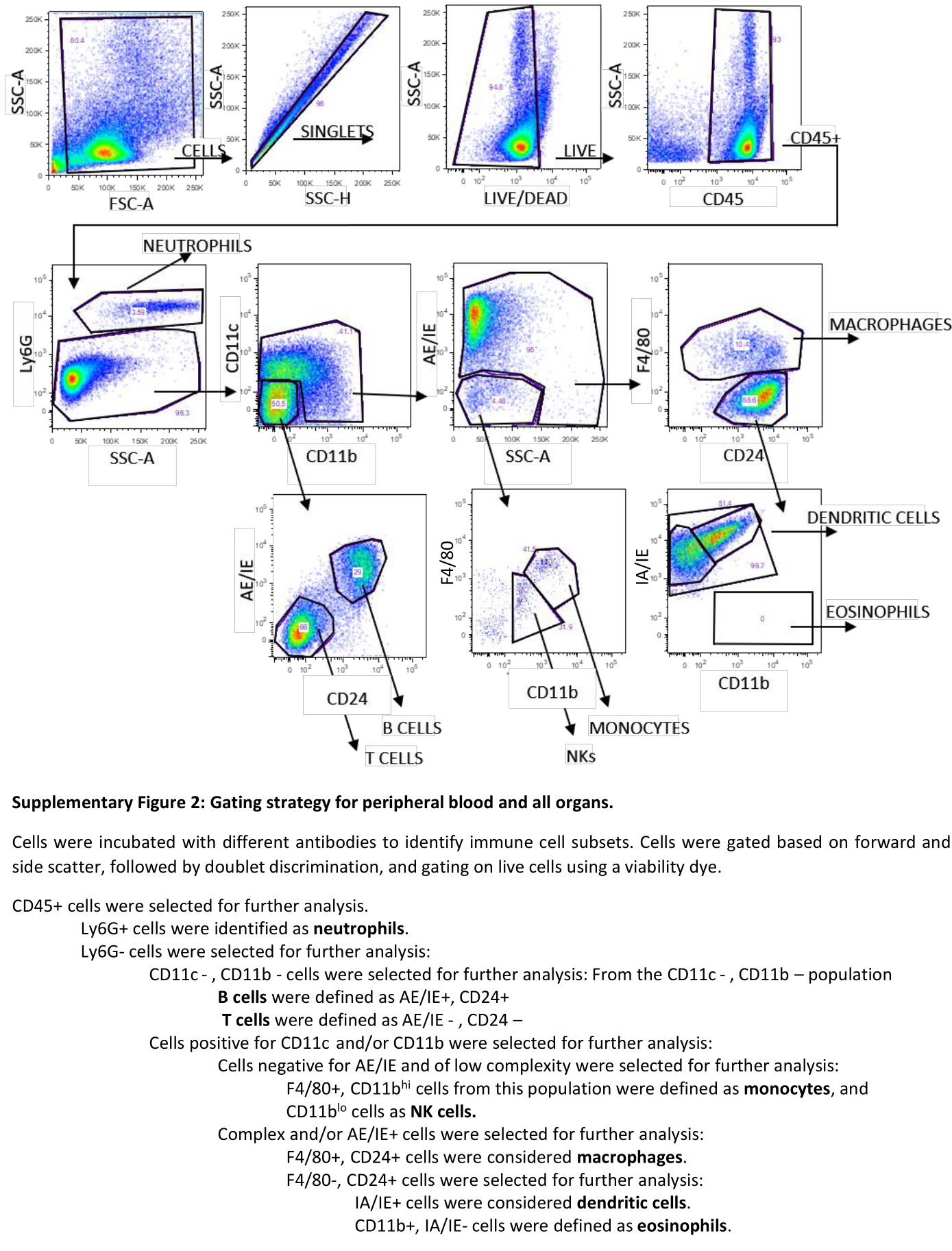

Supplement: Supplementary file 7 [file Image_2.TIFF]

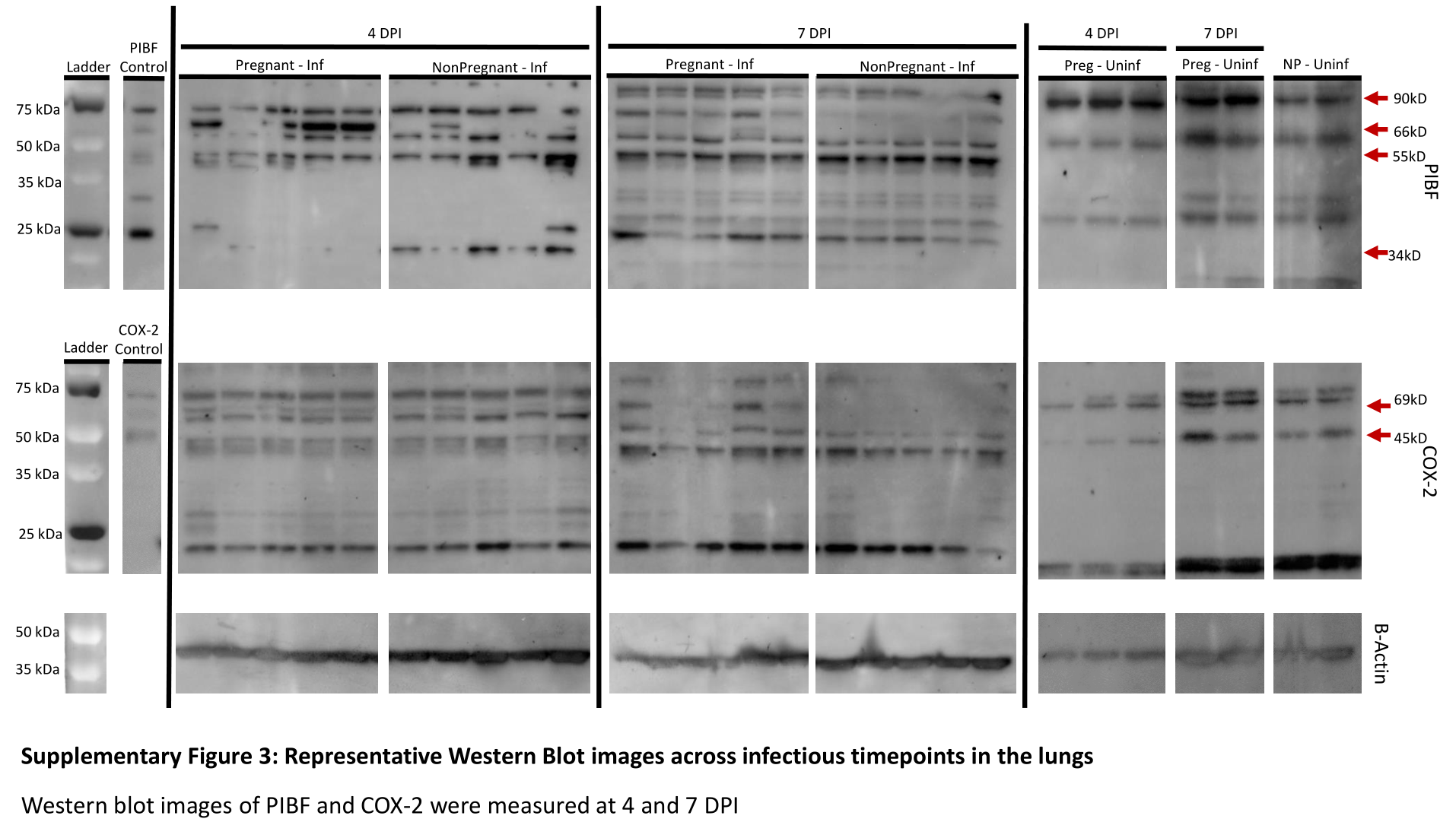

Supplement: Supplementary file 8 [file Image_3.TIFF]

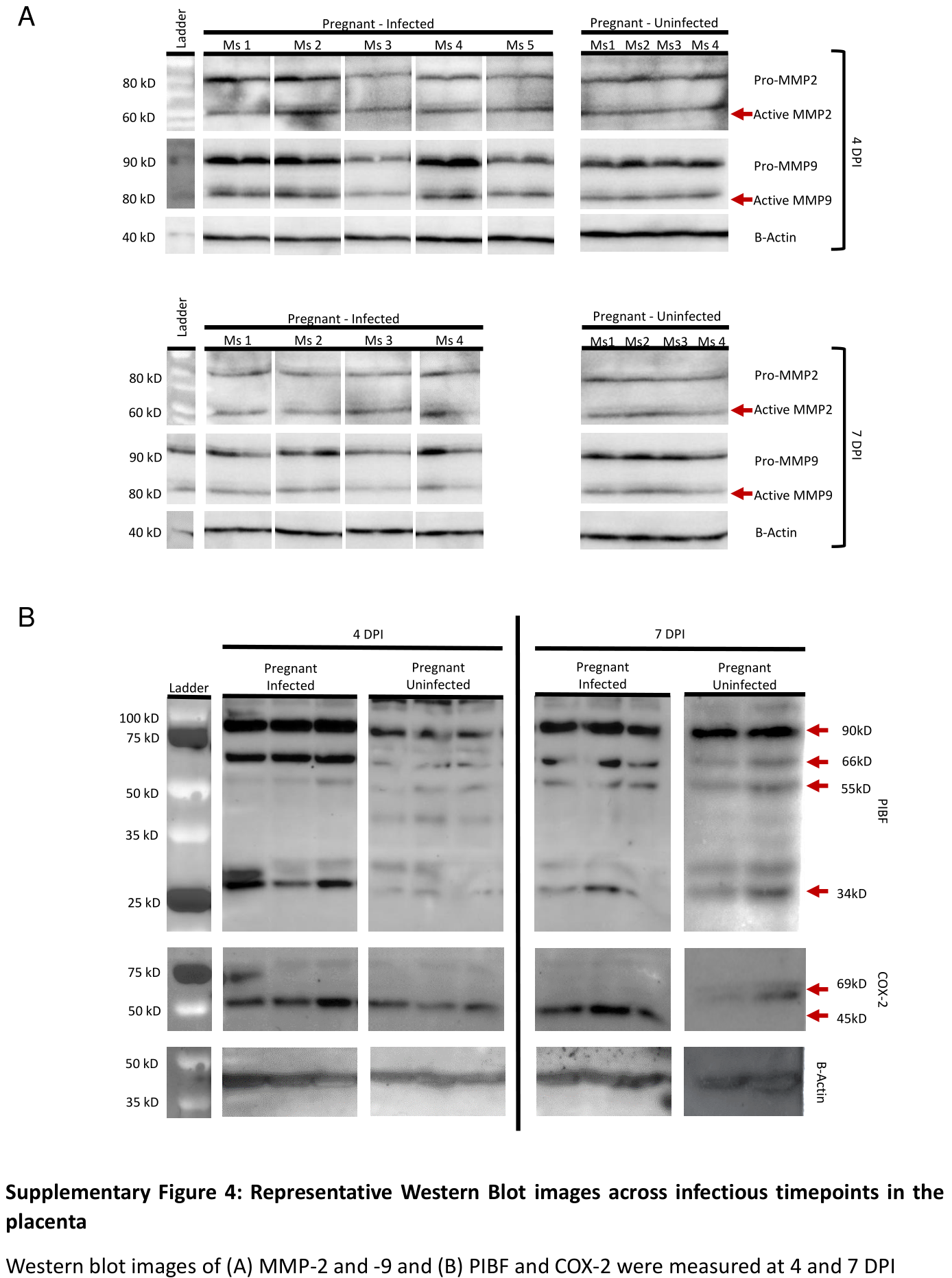

Supplement: Supplementary file 9 [file Image_4.TIFF]
